# Claudin 1–mediated positioning of DC1 to mTECs is essential for maintenance of central tolerance

**DOI:** 10.1084/jem.20250970

**Published:** 2026-01-02

**Authors:** Jiří Březina, Tomáš Brabec, David Machač, Matouš Vobořil, Ondřej Ballek, Jan Pačes, Vojtěch Sýkora, Kristína Jančovičová, Evgeny Valter, Katarína Kováčová, Jasper Manning, Valerie Tahtahová, Adéla Čepková, Martina Dobešová, Jan Dobeš, Jan Kubovčiak, Michal Kolář, Petr Kašpárek, Radislav Sedlacek, Ondřej Štepánek, Jan Černý, Sachiko Tsukita, Bernard Malissen, Graham Anderson, Dominik Filipp

**Affiliations:** 1 Laboratory of Immunobiology, Institute of Molecular Genetics of the Czech Academy of Sciences, Prague, Czech Republic; 2 Department of Cell Biology, Faculty of Science, Charles University, Prague, Czech Republic; 3 Laboratory of Genomics and Bioinformatics, Institute of Molecular Genetics of the Czech Academy of Sciences, Prague, Czech Republic; 4 Czech Centre of Phenogenomics and Laboratory of Transgenic Models of Diseases, Institute of Molecular Genetics of the Czech Academy of Sciences, Vestec, Czech Republic; 5 Laboratory of Adaptive Immunity, Institute of Molecular Genetics of the Czech Academy of Sciences, Prague, Czech Republic; 6 Advanced Comprehensive Research Organization, Teikyo University, Tokyo, Japan; 7 Centre d’Immunologie de Marseille-Luminy, Aix Marseille Universite´, Inserm, CNRS, Marseille, France; 8 Institute of Immunology and Immunotherapy, University of Birmingham, Birmingham, UK

## Abstract

Central tolerance, which relies on the presentation of self-antigens by mTECs and DCs, prevents autoimmunity by eliminating self-reactive T cells. While mTECs produce self-antigens autonomously, DCs acquire them from mTECs via cooperative antigen transfer (CAT). We previously showed that mTEC and DC subsets exhibit preferential pairing in CAT, providing a rationale for the existence of molecular determinants underpinning this pairing and its outcome. Here, we compared the transcriptomes of CAT-experienced and CAT-inexperienced DCs and identified Claudin 1 as a molecule involved in CAT and type 1 DC (DC1) maturation. DC1-specific ablation of Claudin 1 resulted in decreased CAT to late mature DC1s and dramatically diminished DC1 maturation. These phenotypes correlated with the displacement of DC1s from mTECs and their decreased expression of MHCII pathway genes. This translated into impaired Treg selection and clonal deletion, ultimately manifesting in symptoms of multiorgan autoimmunity and shortened lifespan. Collectively, our results identify thymic DC1-derived Claudin 1 as a regulator of immune tolerance.

## Teaser

Březina et al. demonstrate that Claudin 1 enables thymic type 1 dendritic cells to localize within proximity to medullary thymic epithelial cells. This spatial arrangement supports maturation and tolerogenic function of type 1 dendritic cells, thus preventing disruptions in T-cell tolerance.

## Introduction

The vast T-cell receptor (TCR) repertoire of the adaptive immune system would be detrimental to the host if self-reactive T cells were not properly selected (Klein and Petrozziello, 2024). The mechanistic basis for this selection, which occurs largely in the thymic medulla, is the presentation of self-antigens to developing T cells, a process known as central tolerance. Medullary thymic epithelial cells (mTECs) and thymic dendritic cells (DCs) are antigen-presenting cells (APCs), which are instrumental in this process (Klein et al., 2019; Březina et al., 2022). In addition to ubiquitous self-antigens, the murine genome encodes ∼6,500 genes whose products, referred to as tissue-restricted antigens (TRAs) (Sansom et al., 2014), e.g., insulin (Jolicoeur et al., 1994) or enteric defensins (Dobeš et al., 2015), are only found in a limited number of extrathymic tissues (Klein and Petrozziello, 2024). To prevent TRA-targeted autoimmunity, TRAs are also expressed on mTECs, which employ a unique transcriptional machinery that is directed by an unconventional transcriptional modulator, autoimmune regulator (Aire), that mediates ectopic TRA expression (Anderson and Su, 2016). Fragments of TRAs, as well as other generic antigens, are directly presented on mTEC MHC molecules that are recognized by developing self-reactive T cells, leading to clonal deletion (recessive tolerance) or conversion to T regulatory cells (Tregs) (dominant tolerance) (Klein et al., 2019). Interestingly, thymic DCs do not express Aire but instead present TRAs indirectly through TRA acquisition from mTECs (Gallegos and Bevan, 2004; Koble and Kyewski, 2009; Vobořil et al., 2022). This process of directional antigen spreading is referred to as cooperative antigen transfer (CAT) and has been shown to be essential in the reinforcement of both recessive and dominant tolerance (Perry and Hsieh, 2016; Kadouri et al., 2020; Březina et al., 2022).

A subset of mTECs that expresses high levels of MHCII, CD80/86, AIRE, and TRAs is referred to as mTEC^HI^, while another subset, mTEC^LO^, exhibits low expression of the same molecules and limited TRA expression (Danan-Gotthold et al., 2016). Regarding mTEC^HI^, it has been recently shown that these cells give rise to a variety of cell types that mimic the transcriptome and phenotype of tissue-specific stromal cells such as keratinocytes, tuft cells, and microfold cells. These “mimetic cells” could serve alongside mTEC^HI^ as a central reservoir of TRAs for CAT (Kadouri et al., 2020; Michelson et al., 2022; Givony et al., 2023; Březina et al., 2022).

Thymic DCs with their potential to acquire mTEC-derived antigens are represented by two conventional DC lineages, type 1 (DC1) and 2 (DC2) (Perry et al., 2014; Leventhal et al., 2016; Breed et al., 2022). The cells of both lineages are efficient in the acquisition of mTEC-derived antigens but differ in their mTEC subset preferences. In particular, DC1s prefer to uptake antigen from mTEC^HI^ and mimetic cells, which are loaded with TRAs, while the DC2 lineage interacts preferentially with mTEC^LO^ (Vobořil et al., 2022). It is of interest that monocyte-derived thymic CD11c^+^ cells complement DC1 and DC2 by being effective in CAT not only from various mTEC subsets but also from other CD11c^+^ cells (Vobořil et al., 2020, 2022).

Recent studies have described CAT as a complex and highly organized process in which preferential engagement of specific mTEC and DC subsets suggests a deterministic nature of their interaction (Vobořil et al., 2022; Millet et al., 2008; Koble and Kyewski, 2009; Perry et al., 2014; Perry et al., 2018; Kroger et al., 2017; Lancaster et al., 2019; Morimoto et al., 2022; Morimoto et al., 2023). However, the molecules that drive mTEC-to-DC CAT are largely unknown, with the exception of the scavenger receptor CD36, which mediates the terminal phase of CAT, i.e., the “scavenging” of mTEC-derived apoptotic bodies by DC1s (Perry et al., 2018). In this context, CD36 is required for the transfer of surface but not cytoplasmic molecules by CAT, which is indicative of trogocytosis (Schriek and Villadangos, 2023). However, we recently found that the transfer of membrane-bound proteins is a less frequently observed mechanism of CAT to DC1s in comparison with the transfer of cytoplasmic molecules, i.e., the phagocytosis of mTEC apoptotic bodies (Vobořil et al., 2022). Thus, since it appears that CAT is a deterministic process, the molecules (other than CD36) that regulate the mTEC subset-to-DC subset interactions remain to be identified.

Recently, it has been also shown that the engulfment of apoptotic cells within tumors and peripheral organs drives homeostatic maturation of immature DC1s (Maier et al., 2020; Bosteels et al., 2023; Silva-Sanchez et al., 2023; Cummings et al., 2016). Mature DC1s possess superior antigen presentation capability that is accompanied by transcriptomic changes in cholesterol metabolism (Ardouin et al., 2016; Bosteels et al., 2023). Interestingly, several studies in the thymus have also shown that immature DC1s give rise to homeostatic mature DC1s (Ardouin et al., 2016; Oh et al., 2018; Breed et al., 2022; Ashby et al., 2024). We refer to these as activated DC1s (aDC1s) based on their elevated activation status (Vobořil et al., 2022). Hence, our intention was to identify the molecule(s) that regulate preferential pairing within CAT, which leads to DC maturation.

In this study, we found a tight junction protein, CLAUDIN 1, that is encoded by the *Cldn1* gene, which impacts CAT and consequently DC1 maturation. Comparative analysis of single-cell RNA sequencing (scRNAseq) of CAT-experienced and CAT-inexperienced myeloid thymic cells allowed us to design a flow cytometry–based gating strategy to redefine the heterogeneity of thymic DCs and their distinct maturation states. Analysis of 3D light sheet fluorescence microscopy images of the thymic medulla determined that Claudin 1 positions DC1s in direct contact with mTECs. We showed that Claudin 1–deficient DC1s exhibited transcriptomic changes in MHCII pathway genes. As part of this study, we utilized a *Defa6*^*iCre*^*R26*^*TdTOMATO*^ mouse model in which TdTOMATO was expressed in mTECs through Aire-dependent activation of the *Defa6* promoter, thus mimicking the expression of the natural TRA, enteric α-defensin 6 (Dobeš et al., 2015; Vobořil et al., 2022). Infusing bone marrow (BM) from a mouse harboring a conditional deletion of Claudin 1 in the DC1 lineage (Tokumasu et al., 2016; Wohn et al., 2020) into this model, we determined that Claudin 1 is critical for the presence of CAT-experienced late mature DC1s, ensuring proficient indirect TRA presentation to self-reactive T cells. Indeed, by constructing a novel mouse model, *Defa6*^*iCre*^*R26*^*TdT-OVA*^, we demonstrated the impact of Claudin 1 on Treg selection and clonal deletion of model TRA-specific T cells, which subsequently resulted in a break in tolerance.

## Results

### Claudin 1 is upregulated in CAT-experienced DC1s

**Figure 1.**
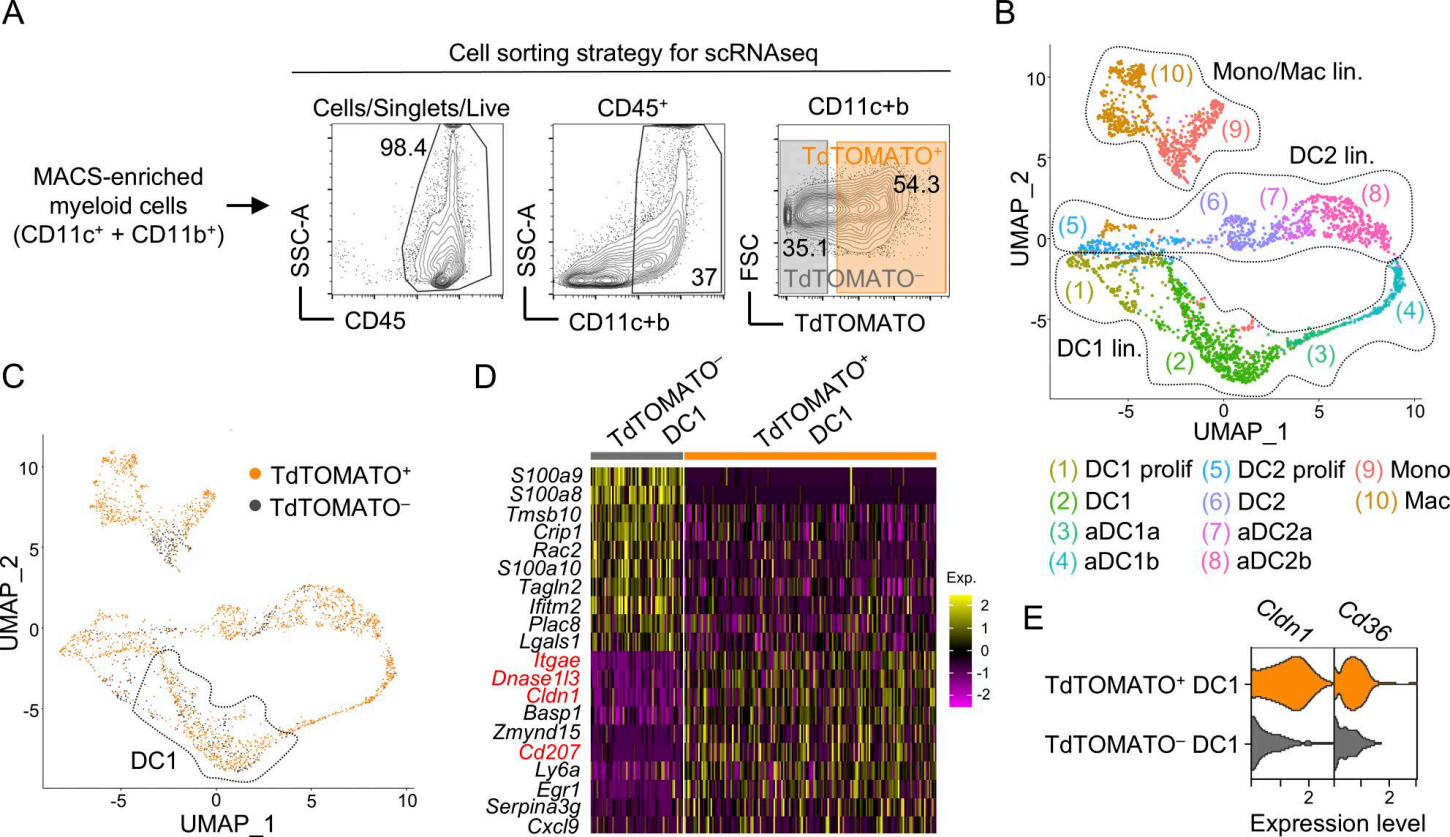
**Claudin 1 is upregulated in CAT-experienced DC1s. (A)** FACS gating strategy used to perform scRNAseq of thymic myeloid cells. Thymic cells were isolated from *Foxn1*^*Cre*^*R26*^*TdTOMATO*^ mice, MACS-enriched for CD11c^+^ and CD11b^+^ cells, and sorted as either TdTOMATO^+^ or TdTOMATO−CD45^+^ CD11c^+^/CD11b^+^ cells. **(B)** UMAP of annotated thymic myeloid cells from scRNAseq excluding subsets shown in red in the legend of Fig. S1 B. Individual cell lineages are demarcated by dotted lines. DC = conventional DC; aDC = activated DC; Mac = macrophages; Mono = monocytes; prolif = proliferating. **(C)** UMAP of scRNAseq corresponding to Fig. 1 B projecting CAT-experienced (orange) and CAT-inexperienced (gray) cells. A dotted line shows the DC1 subset. **(D)** Heat map showing top 10 down- and upregulated genes in CAT-experienced over CAT-inexperienced DC1s. The heat map color scale depicts average log2 fold change. **(E)** Violin plots show the expression of *Cldn1* and *Cd36* by CAT-experienced (orange) and CAT-inexperienced (gray) DC1. All mice were bred on the *B6* background. Littermates were used.

**Figure S1.**
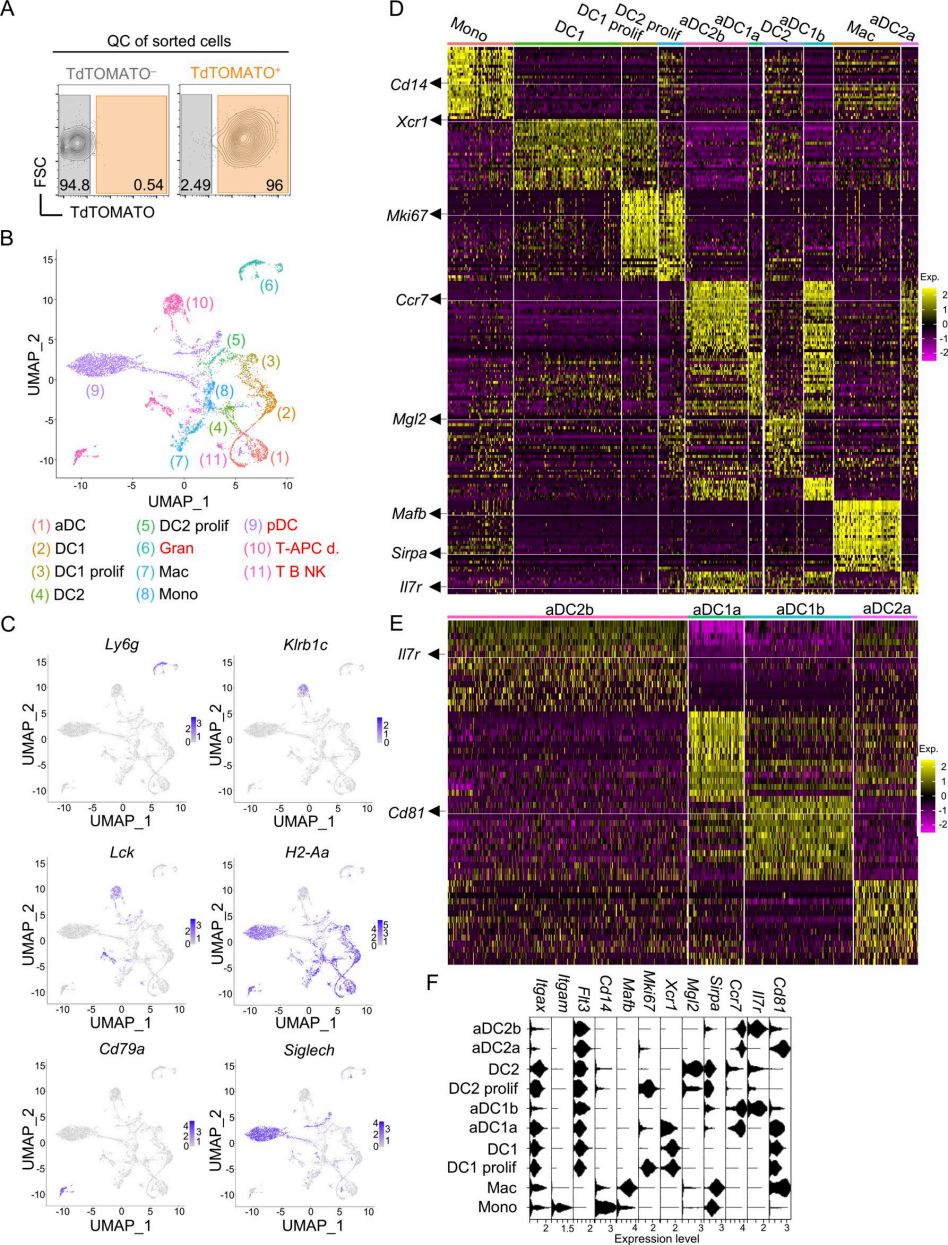
**scRNAseq** **of thymic myeloid APCs. (A)** Quality control contour plots showing the expression level of TdTOMATO in sorted TdTOMATO^−^ and TdTOMATO^+^ thymic myeloid cells. **(B)** UMAP of scRNAseq of all sorted and annotated thymic myeloid cells. Items in red font mark subsets that were excluded from the analysis. DC = conventional DC; aDC = activated DC; Gran = granulocytes; Mac = macrophages; Mono = monocytes; pDC = plasmacytoid DC; T-APC d. = doublets of T cells and APCs; T B NK = T, B, and NK cells; prolif = proliferating. **(C)** UMAP featureplots showing the expression of marker genes of cell subsets that were excluded from scRNAseq analysis. **(D)** Heat map showing up to 25 of the top marker genes of each subset from scRNAseq annotated in Fig. 1 B. The heat map color scale depicts average log_2_ fold change. For better discernibility of the expression profile across all subsets annotated in Fig. 1 B, the selected marker genes are highlighted by arrows and underlined with a white line. **(E)** Heat map showing up to 15 of the top marker genes of each aDC subset from scRNAseq. The heat map color scale depicts average log_2_ fold change. **(F)** Violin plots show the expression of selected marker genes of subsets annotated in Fig. 1 B. All mice were bred on the *B6* background. Littermates were used. Age-matched WT *B6* mice from JAX were used as TdTOMATO^−^ controls to set the TdTOMATO positivity threshold.

To reveal molecules involved in CAT, we compared scRNAseq data of CAT-experienced and CAT-inexperienced myeloid cells from the thymus of 6-wk-old *Foxn1*^*Cre*^*R26*^*TdTOMATO*^ mice, in which the production of TdTOMATO was restricted to TECs (Fig. 1 A and Fig. S1 A) (Vobořil et al., 2020, 2022). Since thymic myeloid cells exhibit significant heterogeneity, we first determined their composition (Fig. S1 B). We excluded the following cell populations from the downstream analysis: granulocytes (Gran; *Ly6g*), T, B, and NK cells (T B NK; *Lck*, *Cd79a*, *Klrb1c*), T-APC doublets (*Lck*, *H2-Aa*), and pDCs (*Siglech*), all of which are not (or marginally) involved in CAT (Fig. S1, B and C) (Kroger et al., 2017; Vobořil et al., 2022). Three distinct lineages of myeloid APCs were found: (1) DC1, (2) DC2, and (3) monocyte/macrophage (Mono, Mac) (Fig. 1 B). We observed previously undescribed heterogeneity regarding the maturation states of DC1 and DC2. Among DC1 and DC2 lineages, we detected *Ccr7*^*−*^ immature DCs (DC1/2), some of which were proliferating *Mki67*^*+*^ cells (DC1/2 prolif) and *Ccr7*^*+*^ mature, activated DCs (aDC1/2). Mature DCs could be further subdivided into early and late mature developmental states (Ardouin et al., 2016; Breed et al., 2022; Bosteels et al., 2023; Bosteels and Janssens, 2025) which we refer to as “a” and “b,” respectively (Fig. 1 B). Accordingly, while in the a state, the lineage-specific markers of DC1 and DC2, *Xcr1* and *Sirpa*, respectively (Fig. S1, D–F), were readily detectable, the b state of the DC2 lineage exhibited a profound decrease in the expression of *Sirpa*. Surprisingly, we found a DC cluster, which lacked both *Xcr1* and *Sirpa* markers, and thus, its origin with respect to the DC1 or DC2 lineages was unclear. Since this *Xcr1*/*Sirpa* double-negative population clustered with aDC1a, we designated this cluster as aDC1b (Fig. 1 B). In addition, our scRNAseq analysis revealed high expression levels of *Cd81* and *Il7r* in aDC1b and aDC2b, respectively, which we consequently used as markers for these subsets (Fig. S1, E and F).

**Figure S2.**
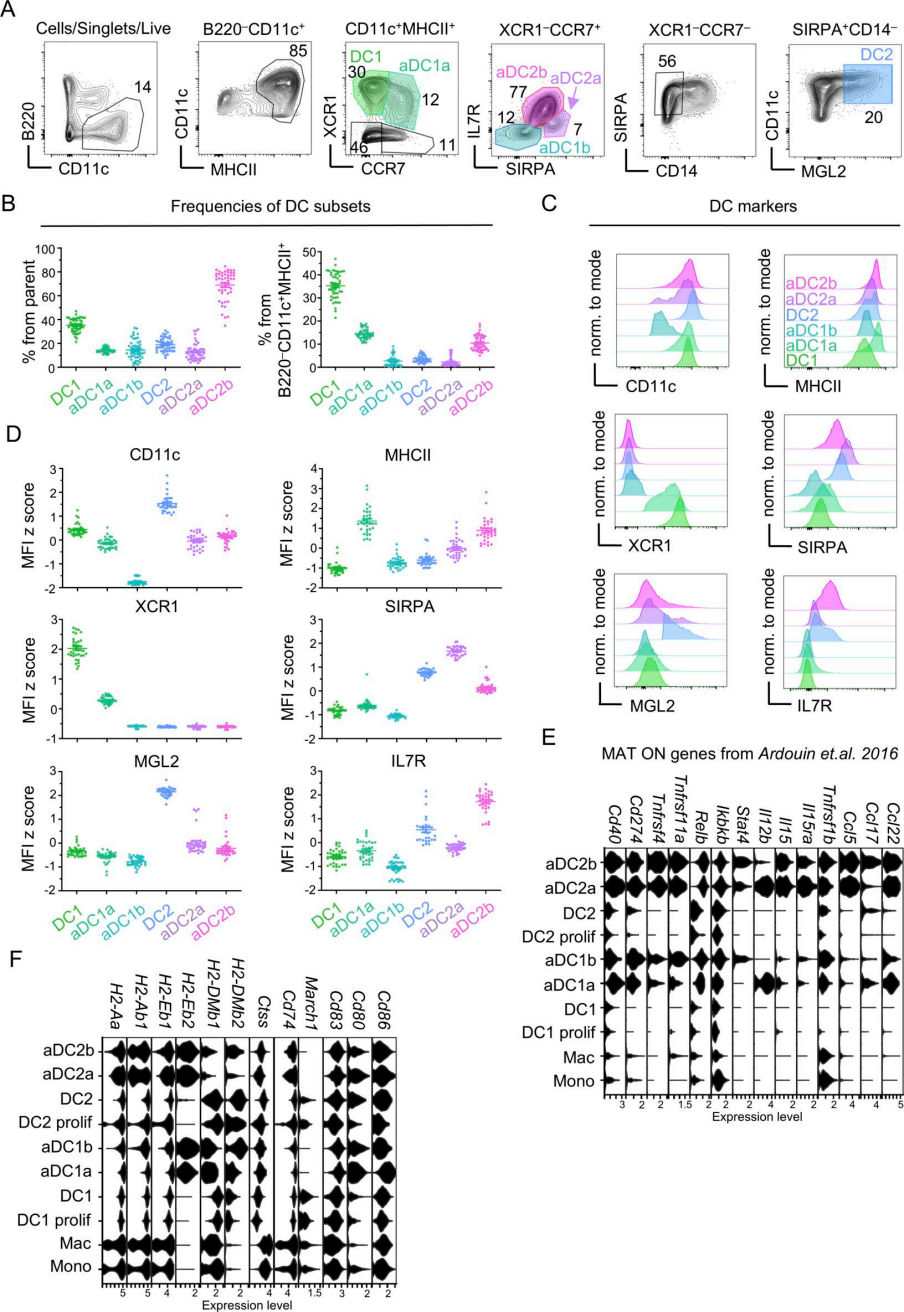
**Expression of marker and antigen presentation–associated genes by thymic DCs. (A)** Flow cytometry gating strategy of thymic DCs based on scRNAseq of thymic myeloid cells. DCs were gated as B220^−^CD11c^+^MHCII^+^ and further distinguished into XCR1^+^CCR7^−^ DC1 and XCR1^+^CCR7^+^ aDC1a. XCR1^−^CCR7^+^ DCs were further separated into SIRPA^High^IL7R^Low^ aDC2a, SIRPA^Low^IL7R^High^ aDC2b, and SIRPA^−^IL7R^−^ aDC1b. XCR1^−^CCR7^−^ cells were then gated as SIRPA^+^MGL2^+^CD14^−^ DC2. The color code of thymic DC subsets defined here is used across all figures. **(B)** Frequencies of DC subsets within parent populations (left panel) and within all B220^–^CD11c^+^MHCII^+^ cells (right panel) (mean ± SEM, *n* = 45–49 mice from seven independent experiments) gated as in Fig. S2 A. **(C)** Histograms depicting the protein expression of DC markers in DC subsets as defined in Fig. S2 A. **(D)** MFI z scores of DC markers within DC subsets related to Fig. S2 C (mean ± SEM, *n* = 29–33 mice from five independent experiments). **(E)** Violin plots show the expression of MAT ON genes taken from Ardouin et al. (2016) within cell subsets annotated in Fig. 1 B. **(F)** Violin plots show the expression of genes involved in MHCII presentation within cell subsets annotated in Fig. 1 B. All mice were bred on the *B6* background. Littermates were used.

Using the selected markers that were identified by scRNAseq, we designed a flow cytometry gating strategy that separated both DC lineages from the monocyte/macrophage lineage (Fig. S2 A) based on the combinatorial expression of *Cd14*, *Sirpa*, and *Mgl2* (Fig. S1 F). As expected, CCR7^+^ aDCs were comprised of aDC1a, aDC1b, aDC2a, and aDC2b, while CCR7^–^ DCs consisted of immature DC1 and DC2 subsets (Fig. S2, A–D). In a previous study, a set of genes referred to as “MAT ON genes” was found to be associated with homeostatic maturation of the thymic DC1 lineage (Ardouin et al., 2016). Remarkably, these genes that encode for chemokines, and costimulatory and checkpoint molecules, such as *Ccl17*, *Ccl22*, *Cd40*, or *Cd274*, are critical in mediating clonal deletion and Treg selection (Hu et al., 2015; Oh et al., 2018; Sharpe and Pauken, 2018). Our scRNAseq analysis confirmed that MAT ON genes are upregulated during maturation of DC1 to aDC1a (Fig. S2 E) (Ardouin et al., 2016). However, we detected a more pronounced upregulation of these genes in the aDC1b subset, which fits with the existence of a late mature state of the DC1 lineage (Fig. S2 E). Consistent with this notion is the fact that both aDC1a and aDC1b expressed high levels of genes involved in the MHCII pathway (Fig. S2 F). It is of note that we also observed the same expression pattern of both MAT ON genes and genes involved in the MHCII pathway along with the DC2 lineage maturation (Fig. S2, E and F).

We recently established that the DC1 lineage is the most effective in CAT (Vobořil et al., 2022). In addition, it has been shown that the thymic DC1 but not the DC2 lineage is specialized for TRA acquisition (Vobořil et al., 2022; Perry et al., 2014; Perry et al., 2018), which is vital for clonal deletion and Treg selection (Klein et al., 2019). Thus, we set out to identify molecules that affect CAT and/or processes that are linked to CAT. Since we hypothesized that gene products that are involved in CAT would be upregulated only in CAT-experienced cells, we conducted a comparative analysis via scRNAseq on both CAT-experienced (TdTOMATO^+^; orange) and CAT-inexperienced (TdTOMATO^−^; gray) cells (Fig. 1 C) and identified the most upregulated genes in TdTOMATO^+^ versus TdTOMATO^−^ cells from the DC1 lineage (Fig. 1 D). Since we observed that CAT occurs in immature DC1s (Vobořil et al., 2022), we focused our search for determinants within this subset (Fig. 1 D). Among the top upregulated genes in CAT-experienced DC1s were *Itgae* (CD103), *Cd207* (Langerin), and *Dnase1l3* (Fig. 1 D) all of which have been shown to be involved in the engulfment of apoptotic cells (Bosteels et al., 2023; Qiu et al., 2009; Sisirak et al., 2016). Among this group of genes, we also identified *Cldn1* (Fig. 1 D), which encodes the tight junction protein CLAUDIN 1 (Furuse et al., 1998). Intriguingly, Claudin 1 has been implicated in the process by which DC1s interact with the intestinal epithelial layer to acquire microbial antigens in the context of the immune response (Rescigno et al., 2001; Farache et al., 2013), a mechanism that has been also described in other epithelial organs such as the lungs and skin (Sung et al., 2006; Kubo et al., 2009). Because mTECs form an epithelial network that is connected by tight junctions (Ichimiya and Kojima, 2006; Sanos et al., 2011), we predicted that in the context of central tolerance, Claudin 1 mediates the interaction of DC1s with mTECs. In fact, while the expression of the scavenger receptor *Cd36* showed only mild enrichment in TdTOMATO^+^ DC1, the enrichment of *Cldn1* was more pronounced (Fig. 1 E). However, it should be noted that CAT-inexperienced DC1s also expressed baseline levels of Claudin 1.

### Lineage tracing and CLAUDIN 1 protein expression in thymic DC1s

**Figure 2.**
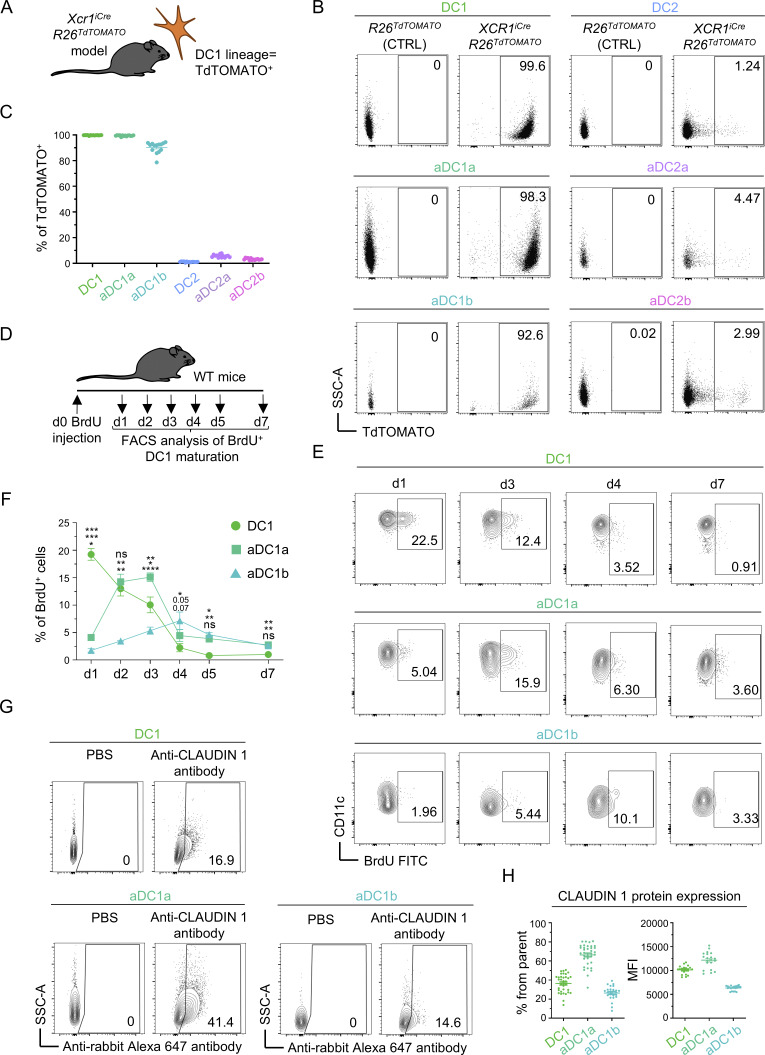
**Lineage tracing and CLAUDIN 1 protein expression in thymic DC1s. (A)** Schematic of the mouse model used for lineage tracing. **(B)** Representative flow cytometry plots show TdTOMATO expression within thymic DC subsets from *XCR1*^*iCre*^*R26*^*TdTOMATO*^ mouse model. **(C)** Frequency of TdTOMATO^+^ cells within DC subsets from Fig. 2 B (mean ± SEM, *n* = 14 mice from three independent experiments). **(D)** Design of BrdU DC1 lineage tracing experiment. **(E)** Representative flow cytometry plots show the frequency of BrdU^+^ cells within DC1 subsets on indicated days after the BrdU administration related to Fig. 2 D. **(F)** Percentage of BrdU^+^ cells within DC1 lineage subsets on indicated days after BrdU administration related to Fig. 2 E (mean ± SEM, *n* = 3–5 mice from two independent experiments). **(G)** Representative flow cytometry plots show CLAUDIN 1 positivity within thymic DC1 subsets. FMO controls are shown. **(H)** Frequency of CLAUDIN 1^+^ cells and MFI of CLAUDIN 1 expression within thymic DC1 subsets related to Fig. 2 G (mean ± SEM, *n* = 20–35 mice from a minimum of three independent experiments). Statistical analysis in F was performed using RM one-way ANOVA with Tukey’s multiple comparisons test, *P ≤ 0.05, **P ≤ 0.01, ***P ≤ 0.001, ****P < 0.0001, ns = not significant. All mice were bred on the *B6* background. Littermates were used as controls. FMO, Fluorescence minus one.

Since aDC1b contain CCR7^+^XCR1^−^SIRPA^−^ DCs whose ontogeny was unclear (Fig. S1, D–F; and Fig. S2, A, C, and D), we fate-mapped thymic DCs using *XCR1*^*iCre*^*R26*^*TdTOMATO*^ mice (Madisen et al., 2010; Wohn et al., 2020). In these mice, TdTOMATO^+^ cells represent DCs with a history of XCR1 expression (Fig. 2 A). Indeed, all thymic DC1 and aDC1a were TdTOMATO^+^ (Fig. 2, B and C). Remarkably, ∼90% of aDC1b were also TdTOMATO^+^, demonstrating that aDC1b that have lost XCR1 expression are members of the DC1 lineage.

To determine the progenitor/progeny relationships within the DC1 lineage, we injected wild-type (WT) mice with BrdU and analyzed its redistribution in defined subsets of the DC1 lineage over a 7-day period (Fig. 2 D). Given that only immature DC1s are capable of proliferation (Fig. S1 F) (Bosteels and Janssens, 2025), BrdU incorporation was observed only in this subset after 24 h. In the ensuing days, the frequency of BrdU^+^ DC1s gradually decreased, while the frequency of BrdU^+^ aDC1a and aDC1b peaked at day 3 and day 4, respectively (Fig. 2, E and F). This is consistent with a scenario whereby DC1 gives rise to early mature aDC1a, a portion of which continues to develop toward late mature aDC1b. When CLAUDIN 1 protein expression was assessed across these subsets, aDC1a exhibited the highest level, while aDC1b exhibited the lowest (Fig. 2, G and H).

### Claudin 1 is involved in CAT and homeostatic DC1 maturation

**Figure 3.**
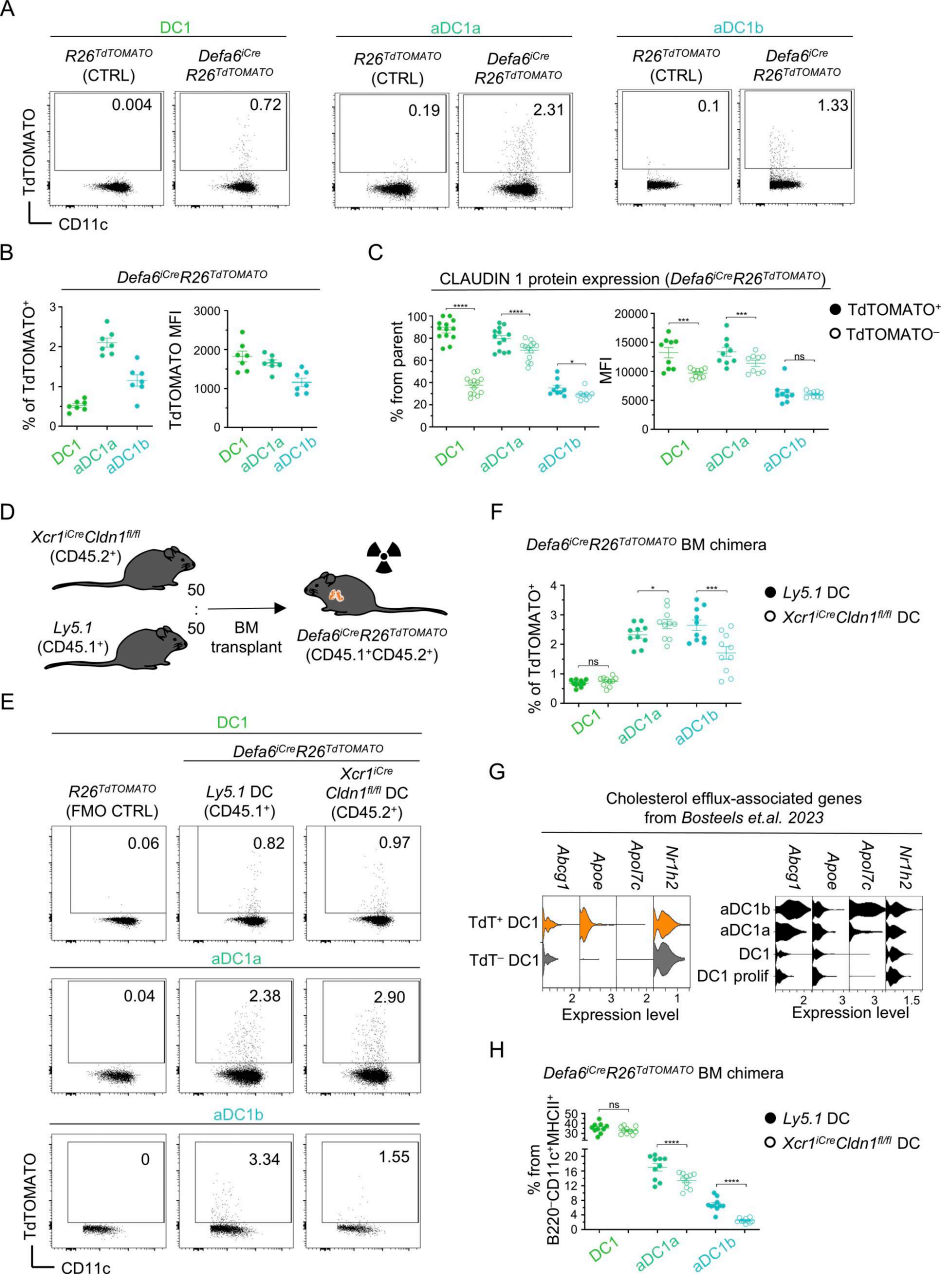
**Claudin 1 is involved in CAT and homeostatic DC1 maturation. (A)** Representative flow cytometry plots show the acquisition of TdTOMATO by thymic DC subsets from *Defa6*^*iCre*^*R26*^*TdTOMATO*^ mice. FMO controls are shown (*R26*^*TdTOMATO*^). **(B)** Frequency of TdTOMATO^+^ cells and MFI of TdTOMATO expression within thymic DC subsets from Fig. 3 A (mean ± SEM, *n* = 7 mice from two independent experiments). **(C)** Frequency of CLAUDIN 1^+^ cells and MFI of CLAUDIN 1 expression within TdTOMATO^+^ and TdTOMATO^−^ DC1 subsets from thymi of *Defa6*^*iCre*^*R26*^*TdTOMATO*^ mice (mean ± SEM, *n* = 9–14 mice from a minimum of three independent experiments). **(D)** Schematic of competitive BM chimera experiment assessing the role of Claudin 1 in CAT. **(E)** Representative flow cytometry plots show the frequency of TdTOMATO^+^ cells within Claudin 1–sufficient (*Ly5.1* BM) and Claudin 1–deficient (*XCR1*^*iCre*^*Cldn1*^*fl/fl*^ BM) DC1 subsets from competitive BM chimeras in Fig. 3 D. FMO controls are shown (*R26*^*TdTOMATO*^ mouse). **(F)** Frequency of TdTOMATO^+^ cells within Claudin 1–sufficient and Claudin 1–deficient DC1 subsets from Fig. 3 E (mean ± SEM, *n* = 10 mice from three independent experiments). **(G)** Violin plots from scRNAseq analysis (Fig. 1) show the expression of cholesterol efflux–associated genes within CAT-experienced (orange) and CAT-inexperienced (gray) DC1 (left panel) and DC1 lineage subsets (right panel). **(H)** Frequency of individual DC1 subsets within thymic DCs from Claudin 1–sufficient (solid circle) and Claudin 1–deficient (empty circle) BM from Fig. 3 D (mean ± SEM, *n* = 10 mice from three independent experiments). Statistical analysis in C, F, and H was performed using paired, two-tailed Student’s *t* test, *P ≤ 0.05, ***P ≤ 0.001, ****P < 0.0001, ns = not significant. All mice were bred on the *B6* background. Littermates were used as controls.

Since we confirmed that DC1, aDC1a, and aDC1b are members of the thymic DC1 lineage, we determined the ability of each subset to acquire TRAs in the *Defa6*^*iCre*^*R26*^*TdTOMATO*^ mouse model. Notably, this model, unlike the *Foxn1*^*Cre*^*R26*^*TdTOMATO*^ model, allowed the tracing of CAT primarily between mTEC^HI^ and the DC1 lineages (Vobořil et al., 2022). The highest frequency of TdTOMATO^+^ cells was detected within aDC1a, an intermediary frequency in aDC1b, and the lowest frequency in DC1 (Fig. 3, A and B). In agreement with our scRNAseq, TdTOMATO^+^ DC1s showed a higher frequency of CLAUDIN 1^+^ cells, as well as a higher level of CLAUDIN 1 expression in comparison with TdTOMATO^−^ cells (Fig. 3 C), further indicating a close relationship between CAT and CLAUDIN 1 expression. In fact, nearly all TdTOMATO^+^ DC1 and aDC1a analyzed expressed CLAUDIN 1.

**Figure S3.**
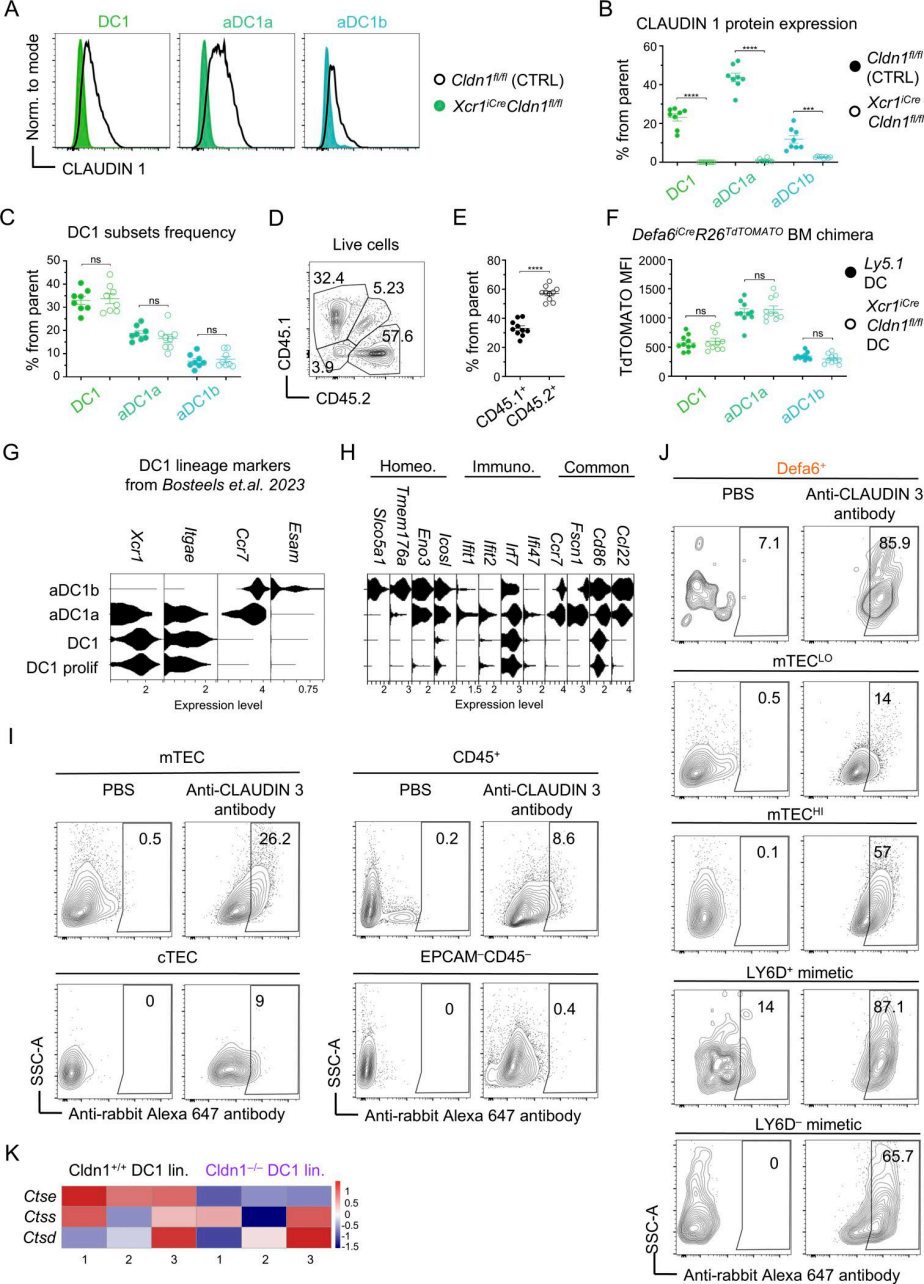
**Mouse models used to study the role of Claudin in CAT. (A)** Histograms show CLAUDIN 1 expression within thymic DC1 subsets from *Cldn1*^*fl/fl*^ (control; black) and *XCR1*^*iCre*^*Cldn1*^*fl/fl*^ (varying shades of green) mice. **(B)** Frequency of CLAUDIN 1^+^ cells within DC1 subsets related to Fig. S3 A (mean ± SEM, *n* = 8 mice from three independent experiments). **(C)** Frequency of DC1 lineage subsets within parent populations from *Cldn1*^*fl/fl*^ (control; solid circle) and *XCR1*^*iCre*^*Cldn1*^*fl/fl*^ (empty circle) mice (mean ± SEM, *n* = 8 mice from three independent experiments). **(D)** Representative flow cytometry plot shows the reconstitution of BMs that give rise to *Ly5.1* DC1 (CD45.1^+^) and *XCR1*^*iCre*^*Cldn1*^*fl/fl*^ DC1 (CD45.2^+^) within BM chimeras from Fig. 3 D. **(E)** Quantification of BM reconstitution from Fig. S3 D (mean ± SEM, *n* = 17 mice from four independent experiments). **(F)** MFI of TdTOMATO expression within Claudin 1–sufficient and Claudin 1–deficient DC1 subsets from Fig. 3, D and E (mean ± SEM, *n* = 10 mice from three independent experiments). **(G)** Violin plots from scRNAseq analysis (Fig. 1) show the expression of DC1 lineage markers used for flow cytometry gating strategy of DC1 subsets in Bosteels et al. (2023). **(H)** Violin plots from scRNAseq analysis (Fig. 1) show the expression of selected marker genes of homeostatic (Homeo.) and immunogenic (Immuno.) maturation and genes associated with both maturation programs (Common) from Bosteels et al. (2023). The subsets analyzed are the same as in Fig. S3 G. **(I)** Representative flow cytometry plots show CLAUDIN 3 positivity within mTECs, cTECs, CD45^+^ cells (hematopoietic cells), and EPCAM^–^CD45^−^ cells (fibroblasts, endothelial cells, etc.). FMO controls are shown. **(J)** Representative flow cytometry plots show CLAUDIN 3 positivity within individual mTEC subsets gated as in Fig. 4 A. FMO controls are shown. **(K)** Heat map showing the relative expression of genes encoding cathepsins across individual sequenced samples. The heat map color scale corresponds to z scores of regularized log data values. The numbers below each column in the heat map correspond to three independent biological replicates, each derived from an individual mouse. Statistical analysis in B and C was performed using unpaired, while statistical analysis in E and F was analyzed by paired, two-tailed Student’s *t* test, ***P ≤ 0.001, ****P < 0.0001, ns = not significant. All mice were bred on the *B6* background. Littermates were used as controls.

In order to determine whether Claudin 1 is a molecular determinant of CAT, we ablated its expression by crossing a *Cldn1*^*fl/fl*^ mouse strain (Tokumasu et al., 2016) with a *XCR1*^*iCre*^ model (Wohn et al., 2020). In *XCR1*^*iCre*^*Cldn1*^*fl/fl*^ mice, Claudin 1 was not detected in the DC1 lineage (Fig. S3, A and B) and the frequency of individual DC1 subsets remained unchanged in comparison with controls (Fig. S3 C). To analyze CAT of TdTOMATO protein, *Defa6*^*iCre*^*R26*^*TdTOMATO*^ mice (CD45.1^+^CD45.2^+^) were sublethally irradiated and reconstituted with a mixture of BM from *XCR1*^*iCre*^*Cldn1*^*fl/fl*^ (CD45.2^+^) and WT (CD45.1^+^) mice at a 1:1 ratio (Fig. 3 D; and Fig. S3, D and E). This experimental setup enabled us to compare the participation of Claudin 1–sufficient versus Claudin 1–deficient DC1 lineage in CAT within a shared thymic microenvironment. We observed that the median fluorescence intensity (MFI) of TdTOMATO was comparable between Claudin 1–sufficient and Claudin 1–deficient cells in all three DC1 lineage subsets (Fig. S3 F), suggesting that Claudin 1 did not affect the quantity of mTEC-derived antigens acquired by individual DCs. Importantly, we found a significant decrease in the frequency of TdTOMATO^+^ aDC1b in Claudin 1–deficient cells when compared to their WT counterparts (Fig. 3, E and F). In contrast to our expectations, we did not observe a decrease in Claudin 1–deficient TdTOMATO^+^ DC1s or aDC1a. In fact, the frequency of TdTOMATO^+^ aDC1a was slightly increased among Claudin 1–deficient cells. Nevertheless, our data show that Claudin 1 is required for efficient CAT to the aDC1b subset, although its deletion results in a relatively mild defect in this process.

Previous studies have shown that engulfment of apoptotic bodies is the primary driver of homeostatic maturation within the DC1 lineage (Cummings et al., 2016; Silva-Sanchez et al., 2023; Maier et al., 2020; Bosteels et al., 2023). We hypothesized that participation in CAT may induce a similar phenotype in thymic DC1s. Indeed, the transcriptional signature that has been published for homeostatic DC1 maturation triggered by apoptotic cell uptake (Bosteels et al., 2023) matches the signature of TdTOMATO^+^ CAT-experienced DC1s and is aligned with the progression of thymic DC1 maturation (Fig. 3 G). In addition, our scRNAseq data showed that in contrast to other thymic DC1 subsets, aDC1b expressed the marker, *Esam* (Fig. S3 G), which has been associated with cholesterol efflux–dependent homeostatic mature splenic DC1s (Bosteels et al., 2023). It is of note that as thymic DC1s matured, distinct sets of genes that are associated with either homeostatic or both homeostatic and immunogenic maturation, but not immunogenic maturation alone, were gradually upregulated (Fig. S3 H) (Bosteels et al., 2023; Bosteels and Janssens, 2025). To directly test whether CLAUDIN 1 has a role in DC1 maturation, we analyzed the DC1 lineage composition in *Defa6*^*iCre*^*R26*^*TdTOMATO*^ BM chimeric mice (Fig. 3 H). Consistent with the involvement of CLAUDIN 1 in DC1 maturation, while there was no effect of Claudin 1 deficiency on the percentage of immature DC1s, the frequency of aDC1a was significantly reduced with an even higher reduction (nearly threefold) in the percentage of aDC1b in comparison with controls.

### Claudin 1 facilitates positioning and contact between DC1s and mTECs

**Figure 4.**
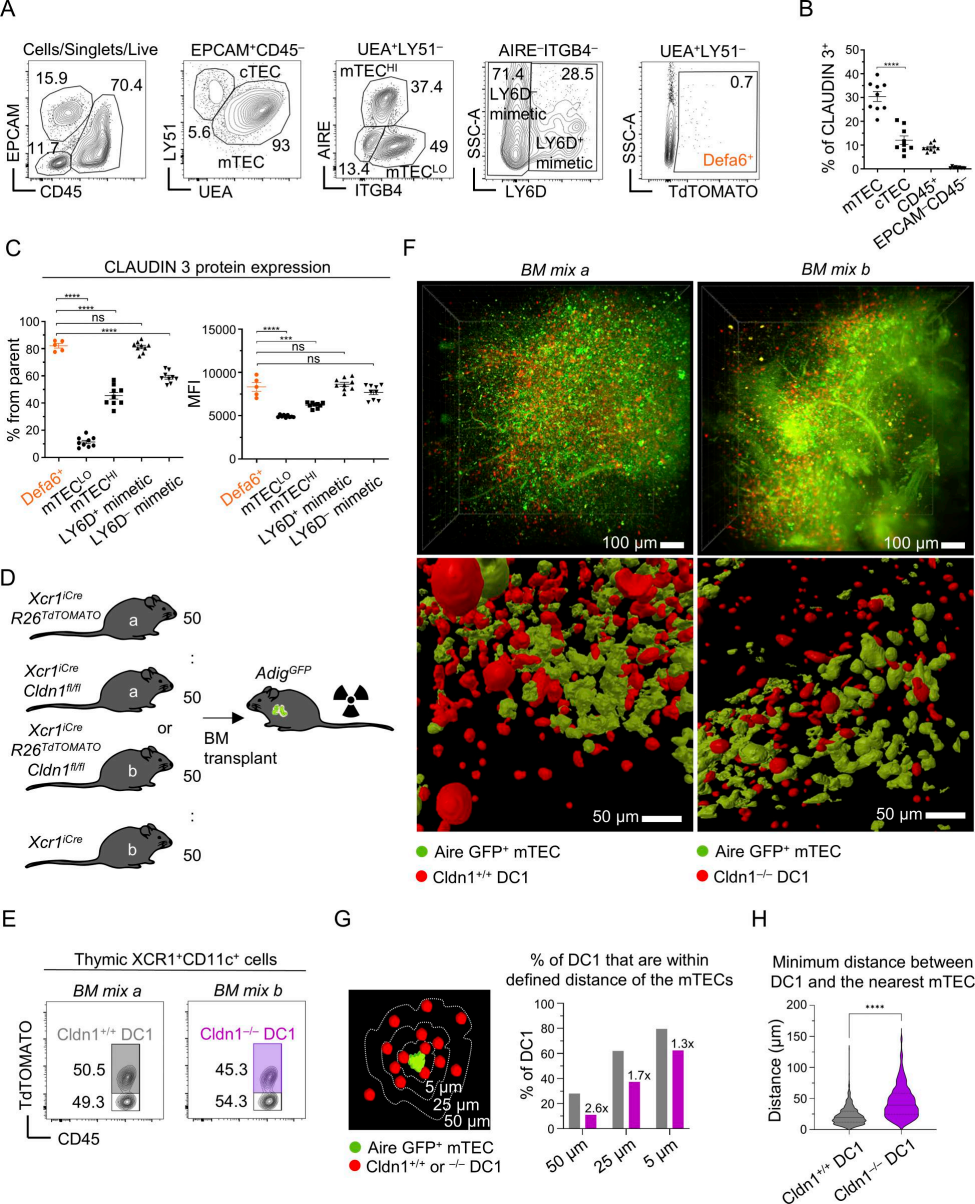
**Claudin 1 facilitates positioning and contact between DC1s and mTECs. (A)** Flow cytometry gating strategy of mTEC subsets. TECs were gated as EPCAM^+^CD45^−^ and further distinguished to LY51^+^UEA^−^ cortical thymic epithelial cells (cTECs) and LY51^−^UEA^+^ mTECs. The latter were further separated into AIRE^+^ITGB4^−^ mTEC^HI^, AIRE^–^ITGB4^+^ mTEC^LO^, and double-negative cells, which were comprised of keratinocyte mimetics (LY6D^+^) and other mimetic cells (LY6D^−^). Defa6^+^ mTECs are color-coded in orange denoting their TdTOMATO positivity. **(B)** Frequency of CLAUDIN 3^+^ cells within cell populations from Fig. S3 I (mean ± SEM, *n* = 9 mice from three independent experiments). **(C)** Frequency of CLAUDIN 3^+^ cells and MFI of CLAUDIN 3 expression within mTEC subsets from Fig. S3 J (mean ± SEM, *n* = 5–9 mice from three independent experiments). **(D)** Schematic of competitive BM chimera used to assess the role of Claudin 1 in positioning of DC1 lineage cells in the proximity of mTECs. Mouse models used as donors of BM are marked by the letters a and b. Note that *Adig*^*GFP*^ recipients obtained BM either from mice a (BM mix a) or b (BM mix b). **(E)** Flow cytometry gating strategy used to analyze the reconstitution of competitive BM chimeras from Fig. 4 D. Thymic CD11c^+^XCR1^+^ cells (DC1s) were gated as in Fig. S4 F and either as TdTOMATO^+^ or TdTOMATO^−^. Note that TdTOMATO^+^ DC1s are Cldn1^+/+^ and TdTOMATO^−^ DC1s are Cldn1^−/−^ in the case of mice receiving BM mix a, and TdTOMATO^+^ DC1s are Cldn1^−/−^ and TdTOMATO^−^ DC1s are Cldn1^+/+^ in the case of mice receiving BM mix b. **(F)** Light sheet fluorescence microscopy images of analogous regions within the thymic medulla of competitive BM chimeras from Fig. 4, D and E. The top images capture the entire medullary compartment imaged. The bottom images visualize segmented objects (red, DC1s; and green, mTEC clusters) within selected regions of the whole 3D images shown above. Separate legends are shown for BM mix a and b. **(G)** Schematic of the analysis of the regions imaged in Fig. 4 F. The imaged area of each mTEC cluster captured was expanded by 50, 25, or 5 μm, and the percentage of DC1s from the total within these expanded clusters was counted. Note that DC1s localized up to 5 μm from mTEC clusters are considered to be in direct contact with mTECs (left panel). The percentage of DC1s that are within a defined distance of mTEC clusters related to Fig. 4 F. The number above the Cldn1^−/−^ DC1 columns indicates the fold change reduction in the percentage of Cldn1^−/−^ DC1 with respect to the percentage of Cldn1^+/+^ DC1 (right panel). **(H)** Violin plots showing minimum distance (μm) between DC1 and the nearest mTEC cluster related to Fig. 4 F. Medians and quartiles are shown (*n* = 2,259 Cldn1^+/+^ DC1s and 531 Cldn1^−/−^ DC1s per representative experiment from a total of two experiments). Statistical analysis in B, C, and H was performed using unpaired, two-tailed Student’s *t* test, ***P ≤ 0.001, ****P < 0.0001, ns = not significant. All mice were bred on the *B6* background. Littermates were used as controls.

Next, we sought to elucidate the mechanistic underpinnings of the relationship between Claudin 1, CAT, and DC1 maturation. Several studies have reported that CLAUDIN 1 forms tight junctions via heterotypic binding to CLAUDIN 3, a lineage marker of Aire-expressing mTECs (Furuse et al., 1999; Daugherty et al., 2007). Therefore, we hypothesized that the binding between these proteins influences the juxtapositioning of DC1s to mTECs. As a starting point, we first assessed the levels of Claudin 3 expression across various thymic and mTEC subsets including mTEC^LO^, mTEC^HI^, Ly6D^+^ (keratinocyte mimetics), and Ly6D^−^ mimetic cells (Fig. 4 A). We also analyzed the mTEC subset that expresses a prototypical Aire-dependent TRA, α-defensin 6 (Defa6^+^) using the *Defa6*^*iCre*^*R26*^*TdTOMATO*^ model (Vobořil et al., 2022). Our results were consistent with the findings of Hamazaki et al. (2007), that mTECs had the highest frequency of Claudin 3 expression (Fig. 4 B and Fig. S3 I) with Aire-independent mTEC^LO^ having the lowest frequency of all mTEC subsets analyzed (Fig. 4 C and Fig. S3 J). Importantly, increasing levels of Claudin 3 expression were detected along the developmental pathway of Aire^+^ mTEC^HI^ into Aire^−^ mimetic cells, suggesting that TRA-loaded mTEC subsets were the main target for CAT by Claudin 1^+^ DC1 subsets. In line with this observation, we detected the highest expression of Claudin 3 in Defa6^+^ mTECs and Ly6D^+^ mimetic cells (Fig. 4 C and Fig. S3 J).

Given the interaction between CLAUDIN 1 on DC1s and CLAUDIN 3 on TRA-enriched mTEC subsets, we tested whether mutual mTEC-DC1 positioning would be affected in the absence of Claudin 1. To visualize and measure the distance between mTECs and DC1s, we prepared two BM mixes that reconstituted a sublethally irradiated *Adig*^*GFP*^ mouse, in which GFP marked Aire-expressing mTECs. These mixes, as depicted in Fig. 4 D, gave rise to WT and Claudin 1–deficient DC1s at a ∼1:1 ratio (Fig. 4 E), in which either WT (BM mix a) or Claudin 1–deficient (BM mix b) DC1s were labeled with TdTOMATO allowing the measurement of their distance from GFP^+^ mTECs.

Using a novel approach, we imaged the thymi of these competitive BM chimeras via light sheet fluorescence microscopy in combination with Clear, Unobstructed, Brain Imaging Cocktail and Computational (CUBIC) clearance of thymic tissue (Susaki et al., 2015), which allowed the imaging of large 3D regions of the thymic medulla (Fig. 4 F, top panel). When we compared the thymic medulla of mice that received either BM mix a or b, we observed that in mix a, TdTOMATO^+^ DC1s (Cldn1^+/+^) were in close contact with the mTEC network. However, in the case of mix b, despite their proximity, TdTOMATO^+^ DC1s (Cldn1^−/−^) did not appear to be in direct contact with mTECs (Fig. 4 F, bottom panel). To quantify proximity, we determined the percentage of TdTOMATO^+^ DC1s that were located within either 5, 25, or 50 μm from mTEC clusters (Fig. 4 G). We observed that in general, the difference in the percentages within a given perimeter decreased as the distance increased. Importantly, in the case of DC1s located within 5 μm of the mTEC clusters, the percentage of WT DC1s (28%) was 2.6 times higher than Claudin 1–deficient DC1s (11%). Given that the 5 μm distance between cells was conducive for direct cell contact and a distance >15 μm significantly reduced the likelihood of contact (Liarski et al., 2014; Guo et al., 2015), our data suggest that Claudin 1 deficiency led to the dislocation of DC1s from mTECs, potentially compromising their cooperative characteristics in the establishment of central tolerance (Herbin et al., 2016). In fact, the minimum distance between Cldn1^−/−^ DC1s and the nearest mTEC cluster was significantly longer compared with that of Cldn1^+/+^ DC1s (Fig. 4 H).

### Claudin 1 is critical for the expression of antigen presentation–associated genes

**Figure 5.**
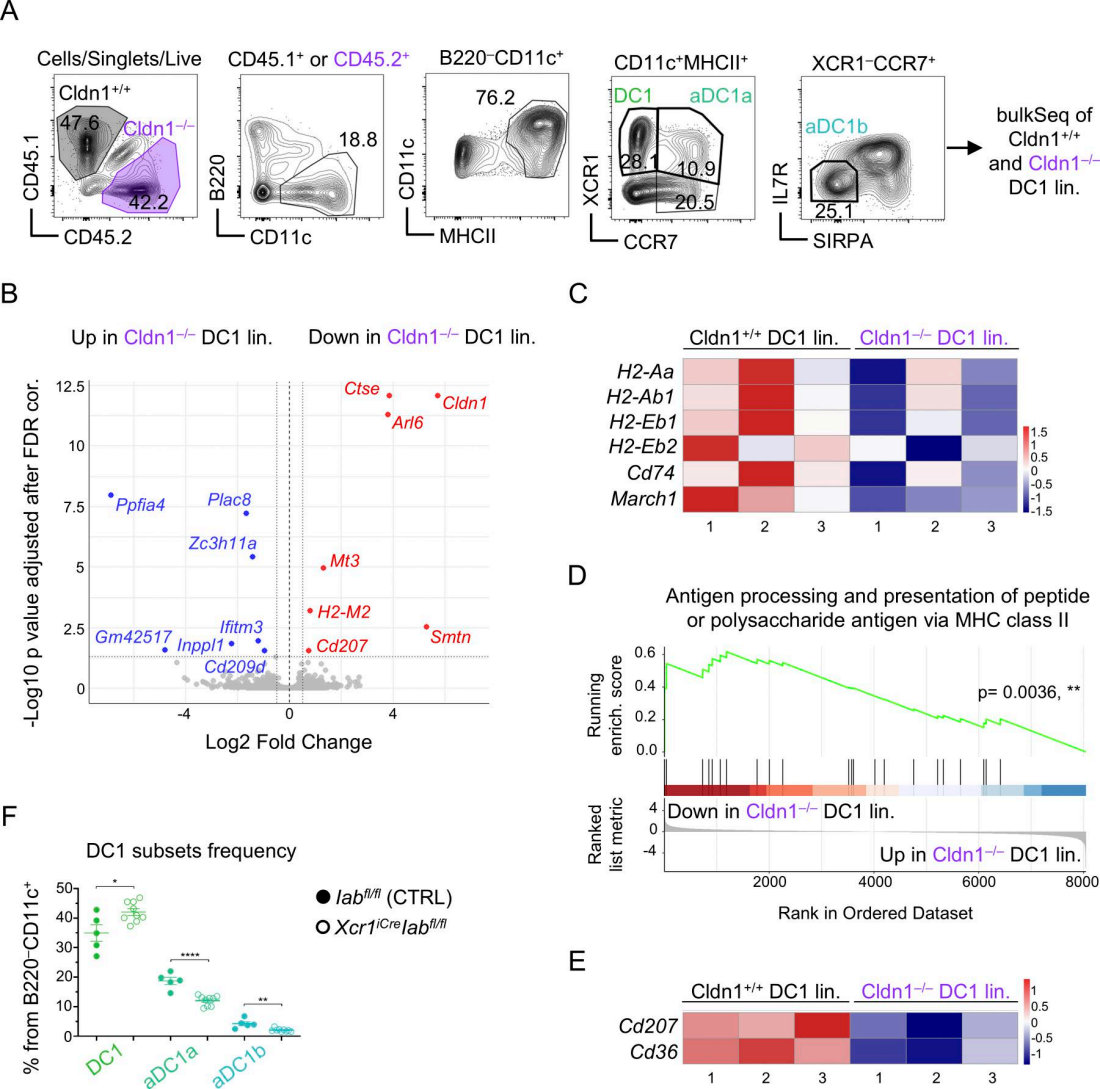
**Claudin 1 is critical for the expression of antigen presentation–associated genes. (A)** Representative FACS gating strategy used to perform bulkSeq of Cldn1^+/+^ and Cldn1^−/−^ thymic DC1 lineage cells isolated from competitive BM chimera described in Fig. 3 D (*n* = 3 mice). Cells were gated as CD45.1^+^ (that included Cldn1^+/+^ DC1 lin.) or CD45.2^+^ (that included Cldn1^−/−^DC1 lin.). Both CD45.1^+^ and CD45.2^+^ cells were further gated as DC1, aDC1a, and aDC1b according to Fig. S2 A. DC1 lineage cells sharing the common origin (CD45.1^+^ or CD45.2^+^) were sorted into a common collection tube and sequenced. Sorting gates are highlighted by thick lines. **(B)** Volcano plot of bulkSeq analysis showing up- and downregulated genes in Cldn1^−/−^ over Cldn1^+/+^ DC1 lineage cells. The threshold of P value adjusted after FDR correction is 0.05 and for log_2_ fold change is 0.5. The baseMean cutoff was set to 10. **(C)** Heat map showing relative expression of genes involved in MHCII presentation across individual sequenced samples. The heat map color scale corresponds to z scores of regularized log data values. The numbers below each column in the heat map correspond to three independent biological replicates, each derived from an individual mouse. **(D)** GSEA of “Antigen processing and presentation of peptide or polysaccharide antigen via MHC class II” pathway between Cldn1^+/+^ and Cldn1^−/−^ DC1 lineage cells. **(E)** Heat map showing the relative expression of *Cd207* and *Cd36* genes in Cldn1^+/+^ and Cldn1^−/−^ DC1 lineage cells. The heat map color scale corresponds to z scores of regularized log data values. The numbers below each column in the heat map correspond to three independent biological replicates, each derived from an individual mouse. **(F)** Frequency of DC1 lineage subsets within B220^–^CD11c^+^ cells from *I-ab*^*fl/fl*^ (control; solid circle) and *XCR1*^*iCre*^*I-ab*^*fl/fl*^ (empty circle) mice (mean ± SEM, *n* = 5–9 mice from two independent experiments). Statistical analysis in F was performed using unpaired, two-tailed Student’s *t* test, *P ≤ 0.05, **P ≤ 0.01, ****P < 0.0001. All mice were bred on the *B6* background. Littermates were used as controls.

To define how Claudin 1 shapes DC1 transcriptional programs, we sorted Cldn1^+/+^ and Cldn1^−/−^ DC1 cells from competitive BM chimeras (Fig. 3 D) and performed bulk RNA sequencing (bulkSeq) (Fig. 5 A). Because Claudin 1 deficiency altered DC1 lineage composition (Fig. 3 H), we analyzed equal numbers of DC1, aDC1a, and aDC1b subsets. As anticipated, Cldn1 was the most downregulated transcript in Cldn1^−/−^ cells, confirming efficient deletion (Fig. 5 B). Strikingly, *Ctse*, encoding the aspartic protease cathepsin E, was also strongly reduced. Cathepsin E has been shown to be critical for antigen processing in the MHCII pathway (Bennett et al., 1992; Zaidi and Kalbacher, 2008), highlighting a potential mechanistic link between Claudin 1 and antigen presentation.

Examining genes involved in antigen acquisition and presentation, we found that other DC-expressed cathepsins (e.g., *Ctss*, *Ctsd*) were unaffected (Fig. S3 K), whereas transcripts encoding MHCII molecules and key MHCII-associated components showed a consistent downward trend in Cldn1^−/−^ DC1 cells (Fig. 5 C). Although downregulation of these genes individually was modest, gene set enrichment analysis (GSEA) revealed significant downregulation of the entire MHCII pathway in Cldn1^−/−^ DC1 lineage cells (Fig. 5 D), indicating a coordinated impairment of antigen presentation machinery.

**Figure S4.**
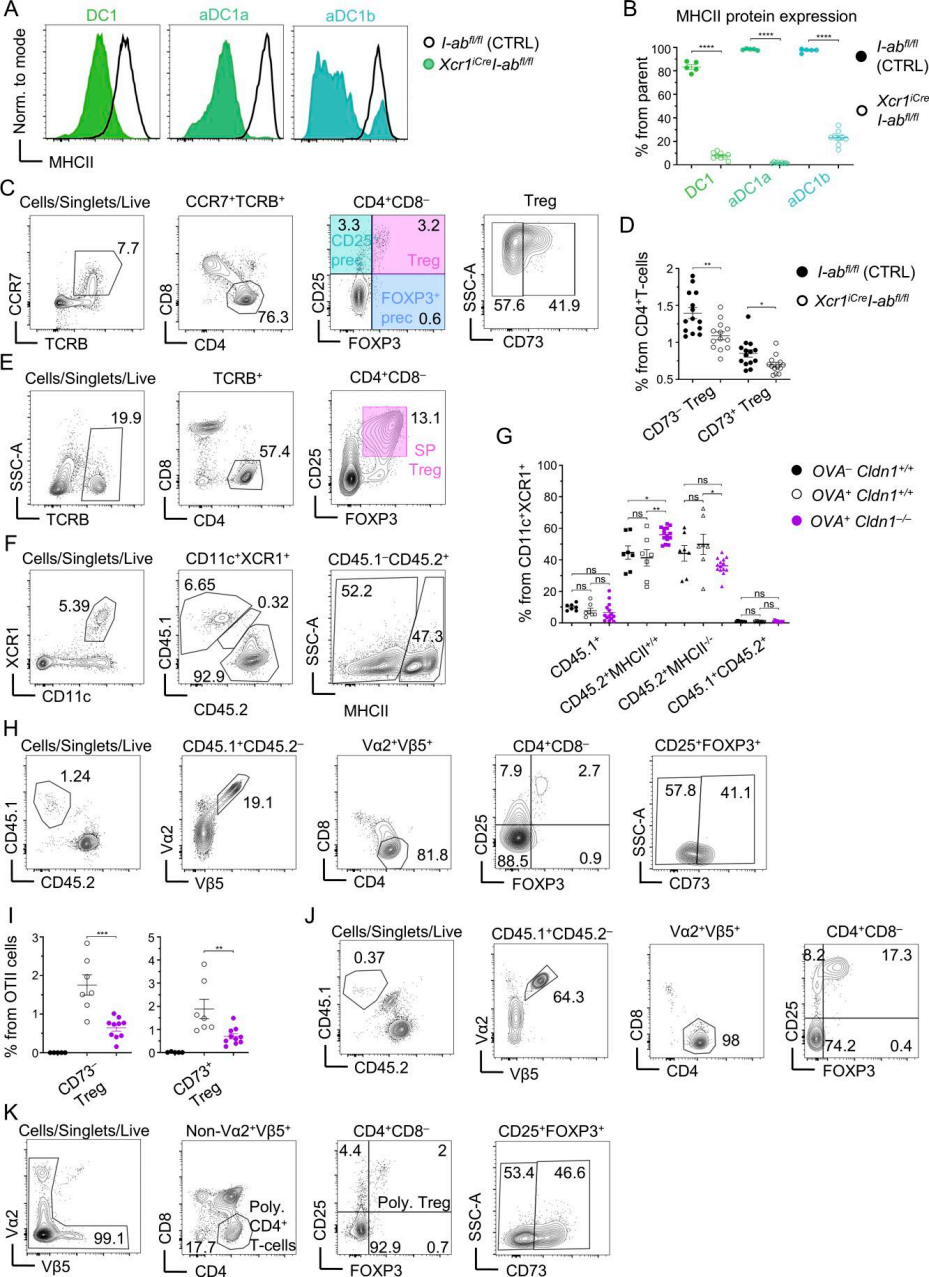
**MHCII-mediated antigen presentation and Claudin 1 expression by DC1 lineage cells are important for the establishment of central tolerance. (A)** Histograms of MHCII expression within DC1 lineage subsets from *I-ab*^*fl/fl*^ (control; black) and *XCR1*^*iCre*^*I-ab*^*fl/fl*^ (varying shades of green) mice. **(B)** Frequency of MHCII^+^ cells within DC1 subsets from *I-ab*^*fl/fl*^ (control; solid circle) and *XCR1*^*iCre*^*I-ab*^*fl/fl*^ (empty circle) mice, related to Fig. S4 A (mean ± SEM, *n* = 5–9 mice from two independent experiments). **(C)** Flow cytometry gating strategy of thymic T cells. T cells were gated as CCR7^+^TCRB^+^ to analyze medullary T cells only. These were gated as CD4^+^ (helper T cells), which were further separated into CD25^+^FOXP3^−^ Treg precursors (CD25^+^ prec), CD25^−^FOXP3^+^ Treg precursors (FOXP3^+^ prec), and CD25^+^FOXP3^+^ Treg. Recirculating Tregs were distinguished from newly generated Tregs as CD73^+^. The color code of thymic T-cell subsets is indicated. **(D)** Frequency of CD73^−^ and CD73^+^ cells within Treg subset from thymi of *I-ab*^*fl/fl*^ (control; solid circle) and *XCR1*^*iCre*^*I-ab*^*fl/fl*^ (empty circle) mice (mean ± SEM, *n* = 13–14 mice from four independent experiments). **(E)** Flow cytometry gating strategy of splenic T cells, which were gated as TCRB^+^CD4^+^ and further separated into Tregs (SP Treg) based on the expression of CD25 and FOXP3. The color code of splenic Tregs is shown. **(F)** Flow cytometry gating strategy to analyze thymic DC1 lineage reconstitution within competitive BM chimeras from Fig. 6, D and E. Note that Cldn1^+/+^MHCII^−/−^ and Cldn1^−/−^MHCII^+/+^ BMs (or Cldn1^+/+^MHCII^+/+^ BM in the controls) were all CD45.2^+^; thus, we quantified the reconstitution of each mixture of these BMs based on the expression of MHCII within thymic DC1 lineage. **(G)** Quantification of reconstitution of thymic DC1 lineage related to Fig. S4 F (mean ± SEM, *n* = 7–14 mice from three independent experiments). The color code is the same as described in Fig. 6 E. **(H)** Flow cytometry gating strategy of OTII thymic T cells. CD45.1^+^ cells were gated as Vα2^+^Vβ5^+^ to obtain OTII cells. These cells were then gated as CD4^+^ and separated into the same populations as in Fig. S4 C and conventional CD25^−^FOXP3^−^OTII cells. **(I)** Frequency of newly generated CD73^−^ (left panel) and recirculating CD73^+^ (right panel) cells within OTII Tregs from thymi of competitive BM chimeras described in Fig. 6, D and E (mean ± SEM, *n* = 5–10 mice from two independent experiments). Color code as in Fig. S4 G. **(J)** Flow cytometry gating strategy of OTII cells from skin-draining lymph nodes. CD45.1^+^ OTII cells were gated as Vα2^+^Vβ5^+^. These were then gated as CD4^+^ and separated into CD25^+^FOXP3^+^ OTII Tregs and conventional CD25^−^FOXP3^−^ OTII cells. **(K)** Flow cytometry gating strategy of developing polyclonal T cells from the thymus. CD4^+^ polyclonal T cells were pregated as non-Vα2^+^Vβ5^+^ to leave out OTII cells from further analysis. These cells were further gated as polyclonal Tregs, which were further distinguished into newly generated (CD73^−^) and recirculating Tregs (CD73^+^). Statistical analysis in G was performed using one-way ANOVA with Tukey’s multiple comparisons test and in B, D, and I was performed using unpaired, two-tailed Student’s *t* test, *P ≤ 0.05, **P ≤ 0.01, ***P ≤ 0.001, ****P < 0.0001, ns = not significant. All mice were bred on the *B6* background. Littermates were used as controls.

We further interrogated antigen acquisition genes and observed modest downregulation of *Cd207* and *Cd36*, which are implicated in apoptotic cell uptake (Bosteels et al., 2023; Qiu et al., 2009; Perry et al., 2018) (Fig. 5, B and E). Langerin, which is encoded by *Cd207*, also facilitates peptide loading onto MHCII (Idoyaga et al., 2008). Given the link between MHCII-mediated presentation and DC maturation (Oh et al., 2018), we analyzed *XCR1*^*iCre*^*I-ab*^*fl/fl*^ mice (Wohn et al., 2020), in which MHCII was selectively ablated in DC1 cells (Fig. S4, A and B). These mice displayed a modest accumulation of immature DC1s with reduced aDC1a and aDC1b subsets (Fig. 5 F), confirming that MHCII-dependent antigen presentation is involved in DC1 homeostatic maturation. Together, these findings indicate that the decreased frequency of mature DC1s in Cldn1^−/−^ mice (Fig. 3 H) is at least partly due to Claudin 1’s role in supporting MHCII pathway function.

### Claudin 1 in DC1 lineage regulates central tolerance

**Figure 6.**
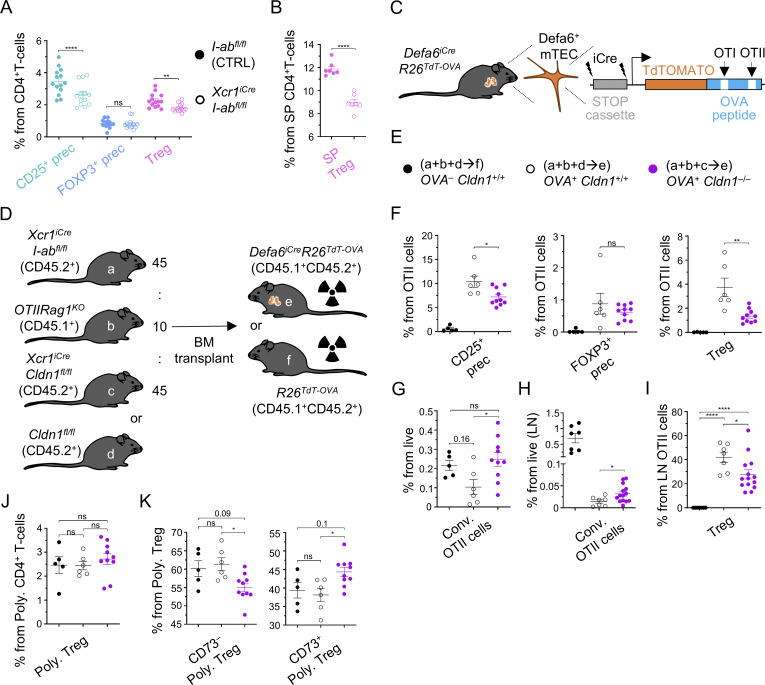
**Claudin 1 in DC1 lineage regulates central tolerance. (A)** Frequency of Tregs and their precursors within CD4^+^ T cells isolated from thymi of *I-ab*^*fl/fl*^ (control; solid circle) and *XCR1*^*iCre*^*I-ab*^*fl/fl*^ (empty circle) mice related to Fig. S4 C (mean ± SEM, *n* = 13–14 mice from four independent experiments). **(B)** Frequency of SP Tregs within CD4^+^ T cells from *I-ab*^*fl/fl*^ (control; solid circle) and *XCR1*^*iCre*^*I-ab*^*fl/fl*^ (empty circle) mice, related to S4E (mean ± SEM, *n* = 7 mice from two independent experiments). **(C)** Schematic of the generated *Defa6*^*iCre*^*R26*^*TdT-OVA*^ model. **(D)** Schematic of donors and recipient genotypes used for competitive BM chimera experiments, which assess the role of Claudin 1 in Treg selection and clonal deletion. Mouse models used are marked by the letters (a–f). Ratio for the preparation of BM mixtures is indicated. **(E)** Symbols used across Figs. 6 and 7 and their supplements for the negative control samples (a+b+d→f, solid black circle), positive control samples (a+b+d→e, empty black circle), and test samples (a+b+c→e, violet circle) are shown. **(F)** Frequency of OTII Tregs and their CD25^+^ and Foxp3^+^ precursors within all OTII cells from thymi of competitive BM chimeras described in Fig. 6, D and E (mean ± SEM, *n* = 5–10 mice from two independent experiments). **(G)** Frequency of conventional OTII cells within all live cells from thymi of competitive BM chimeras described in Fig. 6, D and E (mean ± SEM, *n* = 5–10 mice from two independent experiments). **(H)** Frequency of OTII Tregs within OTII cells from skin-draining lymph nodes of competitive BM chimeras described in Fig. 6, D and E (mean ± SEM, *n* = 7–14 mice from three independent experiments). **(I)** Frequency of conventional OTII cells within live cells from skin-draining lymph nodes of competitive BM chimeras described in Fig. 6, D and E (mean ± SEM, *n* = 7–14 mice from three independent experiments). **(J and K)** Frequency of polyclonal Tregs from polyclonal CD4^+^ T cells (J) and newly generated (CD73^−^) and recirculating Tregs (CD73^+^) from polyclonal Tregs (K) gated as in Fig. S4 K from thymi of competitive BM chimeras described in Fig. 6, D and E (mean ± SEM, *n* = 5–10 mice from two independent experiments). Statistical analysis in A, B, F, and H was performed using unpaired, two-tailed Student’s *t* test and in G, I, J, and K using one-way ANOVA with Tukey’s multiple comparisons, *P ≤ 0.05, **P ≤ 0.01, ****P < 0.0001, ns = not significant. All mice were bred on the *B6* background. Littermates were used as controls.

It has been previously shown that DC1s are important for Treg selection (Perry et al., 2014; Perry et al., 2018), and yet, the involvement of MHCII presentation in this mechanism has not been addressed. To this end, we analyzed the Treg compartment in thymi of *XCR1*^*iCre*^*I-ab*^*fl/fl*^ mice (Fig. S4 C). We found that MHC class II expression by DC1 contributes to the generation of Tregs and CD25^+^ Treg precursors but is ultimately dispensable for the generation of Foxp3^+^ Treg precursors (Owen et al., 2019) (Fig. 6 A). This suggests that antigen presentation by the DC1 lineage governs the development of Tregs from their CD25^+^ but not Foxp3^+^ precursors. Notably, the frequencies of both newly generated CD73^−^ and recirculating CD73^+^ Tregs were also significantly diminished (Fig. S4 D) correlating with the marked reduction of splenic Tregs (Fig. 6 B and Fig. S4 E) (Wohn et al., 2020). Hence, antigen presentation by the DC1 lineage is a crucial contributor to the generation of Tregs within the thymus.

Next, given the preferential pairing of DC1 subsets with TRA-expressing mTEC subsets (Vobořil et al., 2022), we analyzed the function of Claudin 1 in the DC1 lineage in relation to the selection of model TRA-specific T cells. We designed a novel *Defa6*^*iCre*^*R26*^*TdT-OVA*^ mouse model (Fig. 6 C), which contained a TdTOMATO-OVALBUMIN peptide-encoding fusion gene construct (TdT-OVA) within the R26 locus preceded by a STOP cassette flanked by loxP sites. This construct allowed the Cre-driven cell type–specific expression of the TdT-OVA fusion protein. The OVA fragment is comprised of two peptides that are specifically recognized by TCR transgenic OTI and OTII T cells (OTI/II cells). Since this Cre-dependent TdT-OVA system is governed by the promoter of Aire-dependent TRA, α-defensin 6, and is recognized by OTII cells, it is suitable for the study of the generation of OVA-specific thymic Tregs.

To assess the effect of Claudin 1 deficiency in the DC1 lineage on the thymic selection processes, we used mixed BM chimeras in which 10% of the BM was of *OTIIRag1*^*KO*^ origin (Figs. 6, D and E; and Fig. S4, F and G). In this manner, the polyclonal T-cell repertoire was preserved ensuring normal development of the thymus (Akiyama et al., 2008; Hikosaka et al., 2008) while keeping the OTII frequency low and providing optimal conditions for their conversion to Tregs (Bautista et al., 2009). Furthermore, to introduce competition between Claudin 1–deficient and Claudin 1–sufficient DC1 lineages for OVA acquisition, in our test mouse model (*OVA*^*+*^*Cldn1*^*−/−*^), we used a BM mixture composed of Cldn1^+/+^MHCII^−/−^ (from the *XCR1*^*iCre*^*I-ab*^*fl/fl*^ mouse model) and Cldn1^−/−^MHCII^+/+^ DC1 (from the *XCR1*^*iCre*^*Cldn1*^*fl/fl*^ mouse model) at a 1:1 ratio. Presumably, since Claudin 1 is required for juxtaposition of DC1s to TRA-loaded mTECs (Fig. 4), Cldn1^−/−^MHCII^+/+^ DC1 would have limited access to mTECs with OVA in this setting and be positionally outcompeted by Cldn1^+/+^MHCII^−/−^ DC1. Conversely, even though Cldn1^+/+^MHCII^−/−^ DC1 would have access to OVA-producing mTECs, due to the MHCII deficiency, these cells will not be able to present OVA to OTII cells. However, since only the DC1 cells that lack Claudin 1 can present antigens, this experimental design can shed light on the impact of Claudin 1 deficiency on the indirect presentation of mTEC-derived antigens (Březina et al., 2022). Additionally, because Claudin 1 regulates DC1 maturation (Fig. 3), the OVA presentation and presentation of other acquired TRAs by Cldn1^−/−^MHCII^+/+^ DC1 lineage may also be perturbed. It is of note that this model can be used to test the effects on OTII cells and all polyclonal T cells, which represent a majority of T cells in this BM chimeric system. As a reference, we used irradiated mice reconstituted with a BM mixture from MHCII-deficient (Cldn1^+/+^MHCII^−/−^) and Claudin 1–sufficient WT mice (Cldn1^+/+^MHCII^+/+^) on OVA^−^ (*OVA*^*−*^*Cldn1*^*+/+*^, negative control) or OVA^+^ (*OVA*^*+*^*Cldn1*^*+/+*^, positive control) background (Fig. 6, D and E). For flow cytometry analysis, T-cell subsets were gated as depicted in Fig. S4 H.

We detected significantly decreased frequencies of OTII Tregs and OTII CD25^+^ Treg precursors in mice carrying the Claudin 1–deficient (*OVA*^*+*^*Cldn1*^*−/−*^) DC1 lineage in comparison with the *OVA*^*+*^*Cldn1*^*+/+*^ chimeras (Fig. 6 F). It should be noted that the *OVA*^*−*^*Cldn1*^*+/+*^ chimeras did not yield OTII Tregs or their precursors. This was expected since Treg generation requires presentation of their cognate antigen in the thymus (Malchow et al., 2016). Analogous to the experiments with *XCR1*^*iCre*^*I-ab*^*fl/fl*^ mice (Fig. 6 A), the frequencies of OTII Foxp3^+^ Treg precursors were comparable between *OVA*^*+*^*Cldn1*^*+/+*^ and *OVA*^*+*^*Cldn1*^*−/−*^ chimeras (Fig. 6 F). In addition, the frequencies of both newly generated CD73^−^ and recirculating CD73^+^ OTII Tregs were reduced (Fig. S4 I) as in *XCR1*^*iCre*^*I-ab*^*fl/fl*^ mice (Fig. S4 D), indicating that defects in central tolerance were projected to the immune periphery. We also observed a slight increase in the frequency of conventional CD25^−^Foxp3^−^ OTII cells in the thymi of *OVA*^*+*^*Cldn1*^*−/−*^ chimeras in comparison with *OVA*^*+*^*Cldn1*^*+/+*^ chimeras, suggesting incomplete clonal deletion (Fig. 6 G). Since Claudin 1 deficiency affected both Treg selection and clonal deletion, we analyzed its impact on OTII cells in skin-draining lymph nodes (Fig. S4 J). Indeed, we detected a higher frequency of conventional OTII cells in *OVA*^*+*^*Cldn1*^*−/−*^ chimeras (Fig. 6 H) and a reduction in the frequency of lymph node Tregs in comparison with the *OVA*^*+*^*Cldn1*^*+/+*^ chimeras (Fig. 6 I), suggesting that both dominant and recessive central tolerance mechanisms were impaired in the absence of Claudin 1.

The fact that a majority of the T-cell repertoire of our chimeras was polyclonal (Fig. 6 D) prompted us to analyze Treg frequencies from developing polyclonal CD4^+^ T cells (Fig. 6 J and Fig. S4 K). While these frequencies were comparable among all chimera variants analyzed, *OVA*^*+*^*Cldn1*^*−/−*^ mice exhibited a reduced frequency of newly generated Tregs in the thymus that was compensated by an increased frequency of recirculating Tregs (Fig. 6 K). Taken together, these data suggest that Claudin 1 regulates the parameters of the thymic DC1 lineage that are critical for its tolerogenic functions and acts as an essential component of central tolerance impacting clonal deletion and Treg selection.

### Claudin 1 deficiency in the DC1 lineage leads to break in tolerance and premature death

**Figure 7.**
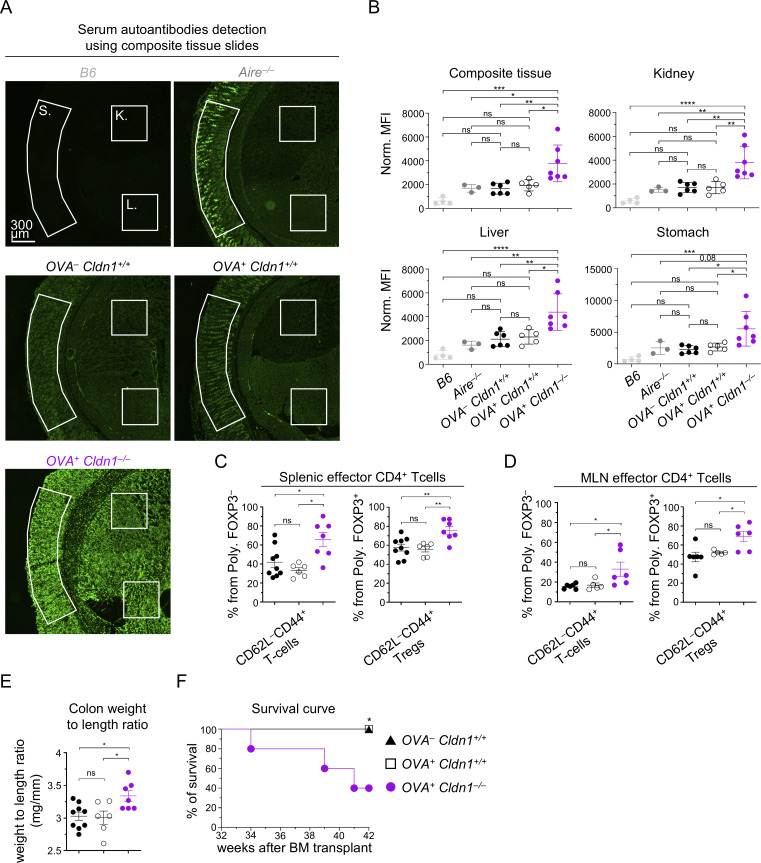
**Claudin 1 deficiency in the DC1 lineage leads to break in tolerance and premature death. (A)** Detection of autoantibodies from sera of BM chimeras from Fig. 6, D and E, using composite tissue slides. Serum autoantibody levels were quantified by the MFI of the secondary anti-mouse antibody conjugated to Alexa 555 (shown in green) across the entire image and from the regions demarcated by white lines corresponding to the individual tissues (K = kidney; L = liver; and S = stomach) that make up the composite slide. 6-wk-old *B6* WT and 30-wk-old *Aire*^*−/−*^ mice on *BALB/c* background were used as negative and positive controls, respectively. Note that at the time of serum harvest, the competitive BM chimeras were 30–40 wk after BM transfer. Slides were imaged using the Tile Scan function in LAS X software (Leica) with a 20% overlap between adjacent tiles, which were subsequently stitched to generate the final composite images. **(B)** Normalized MFI of serum autoantibody detection within the entire image of the composite tissue and individual tissues from Fig. 7 A (mean ± SEM, *n* = 3–7 mice from two independent experiments). **(C and D)** Frequency of effector memory CD62L^−^CD44^+^ cells within polyclonal conventional CD4^+^ T cells (Poly. FOXP3^−^) and Tregs (Poly. FOXP3^+^) gated as in Fig. S5 A, isolated from MLNs (C) and spleens (D) of BM chimeras from Fig. 6, D and E, around 40 wk after BM transfer (*n* = 5–9 mice from two independent experiments). **(E)** Weight-to-length ratio of colons isolated from BM chimeras from Fig. 6, D and E, around 40 wk after BM transfer (*n* = 6–9 mice from two independent experiments). **(F)** Survival curve of BM chimeras from Fig. 6, D and E, (*n* = 5 mice from two independent experiments). Color code as in Fig. 6 E. Note that two *OVA*^*+*^*Cldn1*^*−/−*^ mice died in the first experiment and one *OVA*^*+*^*Cldn1*^*−/−*^ mouse died in the second experiment. Statistical analysis in B–E was performed using one-way ANOVA with Tukey’s multiple comparisons test and in F using the log-rank (Mantel–Cox) test, *P ≤ 0.05, **P ≤ 0.01, ***P ≤ 0.001, ****P < 0.0001, ns = not significant. All mice were bred on the *B6* background except for *Aire*^−/−^ mice, which were bred on the *BALB/c* background. Littermates were used as controls. In A and B, 6-wk-old WT *B6* mice from JAX were used as negative controls and ∼30-wk-old *Aire*^*−/−*^ mice were used as positive controls. MLNs, mesenteric lymph nodes.

Since Claudin 1 is required for the induction of central tolerance, we investigated whether its absence in DC1s would lead to autoimmunity. To this end, we collected sera from competitive BM chimeric mice (Fig. 6, D and E) 30–40 wk after transplantation and analyzed the presence of autoantibodies against kidney, liver, and stomach on composite tissue slides (Fig. 7 A). Young WT mice and old *Aire*^−/−^ mice (both nonirradiated) were used as negative and positive controls, respectively. In all three chimeric variants, we detected reproducible production of autoantibodies in comparison with the negative control (Fig. 7, A and B), which is likely a consequence of mouse irradiation (Alawam et al., 2021) and implementation of the *XCR1*^*iCre*^*I-ab*^*fl/fl*^ mouse model (Figs. 5 and 6). However, while the serum autoantibody levels of the Claudin 1–sufficient chimeras (*OVA*^−^*Cldn1*^*+/+*^ and *OVA*^*+*^*Cldn1*^*+/+*^) were comparable to the positive control (old *Aire*^−/−^ mice) across the entire composite tissue images and in individual organs, *OVA*^*+*^*Cldn1*^*−/−*^ chimeras showed the highest autoantibody levels in all tissues analyzed (Fig. 7, A and B).

**Figure S5.**
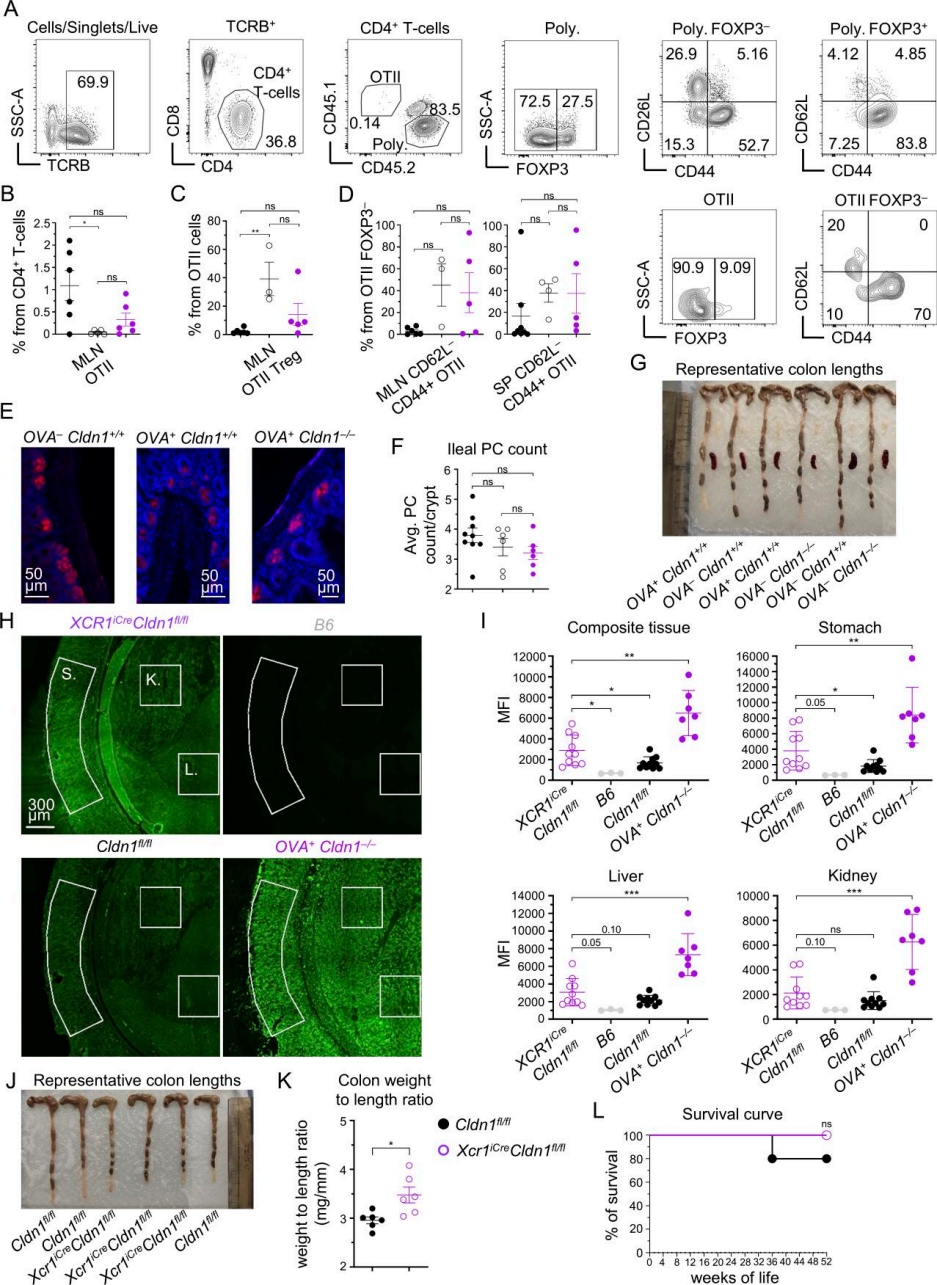
**Autoimmune manifestations of Claudin 1–deficient mice. (A)** Representative flow cytometry gating strategy of activated T cells from MLN and spleen. CD4^+^ T cells were gated as TCRB^+^CD4^+^CD8^−^ and further distinguished using congenic markers into OTII (CD45.1^+^CD45.2^−^) and polyclonal (Poly; CD45.1^−^CD45.2^+^) cells. Both OTII and polyclonal cells were further distinguished into conventional T cells (FOXP3^−^) and Tregs (FOXP3^+^). These populations were analyzed for CD62L and CD44 expression, with CD62L^−^CD44^+^ cells representing effector memory T cells. **(B and C)** Frequency of OTII cells within CD4^+^ T cells (B) and OTII Tregs within OTII cells (C) isolated from MLN of BM chimeras from Fig. 6, D and E, around 40 wk after BM transfer, gated as in Fig. S5 A (mean ± SEM, *n* = 3–6 mice from two independent experiments). **(D)** Frequency of effector memory CD62L^−^CD44^+^ cells within conventional OTII cells (FOXP3^−^) isolated from MLN (left panel) and spleen (right panel) of BM chimeras from Fig. 6, D and E, around 40 wk after BM transfer gated as in Fig. S5 A (mean ± SEM, *n* = 3–8 mice from two independent experiments). **(E)** Representative immunofluorescence images of ileal PCs stained by lysozyme (red) and DAPI (blue) of BM chimeras from Fig. 6, D and E, around 40 wk after BM transfer. **(F)** Quantification of average PC count per crypt within ilea of BM chimeras from Fig. 6, D and E, around 40 wk after BM transfer related to Fig. S5 E (mean ± SEM, *n* = 6–9 mice from two independent experiments). **(G)** Representative colon lengths of BM chimeras from Fig. 6, D and E, around 40 wk after BM transfer. **(H)** Detection of autoantibodies from sera of *XCR1*^*iCre*^*Cldn1*^*fl/fl*^ mice and age-matched *Cldn1*^*fl/fl*^ controls with an average age of ∼35 wk at the serum harvest. Serum autoantibody levels were quantified by the MFI of the secondary anti-mouse antibody conjugated to Alexa 555 (shown in green) across the entire image and from the regions demarcated by white lines corresponding to individual tissues (K = kidney; L = liver; and S = stomach) that make up the composite slide. 6-week-old B6 WT and ∼40-wk-old *OVA*^*+*^*Cldn1*^−/−^ competitive BM chimeras from Fig. 6, D and E, were used as negative and positive controls, respectively. Slides were imaged using the Tile Scan function in LAS X software (Leica) with a 20% overlap between adjacent tiles, which were subsequently stitched to generate the final composite images. **(I)** MFI of serum autoantibody detection within the entire image of the composite tissue and individual tissues from Fig. S5 H (mean ± SEM, *n* = 3–10 mice from two independent experiments). **(J)** Representative colon lengths of *XCR1*^*iCre*^*Cldn1*^*fl/fl*^ mice and age-matched *Cldn1*^*fl/fl*^ controls with an average age of ∼35 wk. **(K)** Weight-to-length ratio of colons isolated from *XCR1*^*iCre*^*Cldn1*^*fl/fl*^ mice and age-matched *Cldn1*^*fl/fl*^ controls with an average age of ∼35 wk (*n* = 6 mice from two independent experiments). **(L)** Survival curve of *XCR1*^*iCre*^*Cldn1*^*fl/fl*^ mice and age-matched *Cldn1*^*fl/fl*^ controls (*n* = 5–7 mice from two independent experiments). Color code as in Fig. S5 K. Statistical analysis in B–D and F was performed using one-way ANOVA with Tukey’s multiple comparisons test, in I and K using unpaired, two-tailed Student’s *t* test, and in L using the log-rank (Mantel–Cox) test, *P ≤ 0.05, **P ≤ 0.01, ***P ≤ 0.001, ns = not significant. All mice were bred on the *B6* background. Littermates were used as controls. In H and I, 6-wk-old WT *B6* mice from JAX were used as negative controls and ∼4-wk-old competitive BM chimeras from Fig. 6, D and E were used as positive controls. MLN, mesenteric lymph node.

While conventional OTII cells and OTII Tregs were still present in the immune periphery up to 40 wk after BM transplantation (Fig. S5, A–C), they were not enriched in the effector CD62L^−^CD44^+^ phenotype (Smigiel et al., 2014) in the mesenteric lymph node or in the spleen in *OVA*^*+*^*Cldn1*^*−/−*^ chimeras, suggesting that they were not responsible for the development of their autoimmune phenotype (Fig. S5 D). Also, Paneth cells (PCs) as the only cells of the immune periphery with an active *Defa6* promoter (the driver of OVA in *OVA*^*+*^*Cldn1*^*+/+*^ and *OVA*^*+*^*Cldn1*^*−/−*^ chimeras) showed similar numbers in the ileum of all BM chimeras (Fig. S5, E and F), further supporting the negligible role of OTIIs in the observed autoimmunity.

On the other hand, the polyclonal CD4^+^ T cells showed an increase in the effector CD62L^−^CD44^+^ phenotype in both splenic conventional T cells and splenic Tregs of *OVA*^*+*^*Cldn1*^*−/−*^ chimeras in comparison with controls (Fig. 7 C and Fig. S5 A). Moreover, we detected the same trend within polyclonal CD4^+^ T cells from mesenteric lymph nodes of *OVA*^*+*^*Cldn1*^*−/−*^ chimeras (Fig. 7 D and Fig. S5 A), indicating inflammation in the intestines. In fact, when compared to controls, a significant increase in colon weight-to-length ratio in the *OVA*^*+*^*Cldn1*^*−/−*^ chimeras (Fig. 7 E and Fig. S5 G), which is a telltale sign of colitis, was observed. Remarkably, this systemic break in tolerance correlated with the premature death of Claudin 1–deficient chimeras (*OVA*^*+*^*Cldn1*^*−/−*^) (Fig. 7 F). Specifically, while all *OVA*^−^*Cldn1*^*+/+*^ and *OVA*^*+*^*Cldn1*^*+/+*^ chimeric mice remained viable 42 wk after BM transplantation, 60% of the *OVA*^*+*^*Cldn1*^*−/−*^ chimeras did not survive beyond this time.

To determine whether the observed autoimmune phenotypes could occur independently of BM transplantation and competition between Claudin 1–sufficient and Claudin 1–deficient DC1, we analyzed nonchimeric *XCR1*^*iCre*^*Cldn1*^*fl/fl*^ mice for signs of autoimmunity. Sera collected from age-matched *Cldn1*^*fl/fl*^ and *XCR1*^*iCre*^*Cldn1*^*fl/fl*^ mice (average age ∼35 wk) were examined for autoantibodies using composite tissue slides with young WT mice and *OVA*^*+*^*Cldn1*^*−/−*^ competitive BM chimeras serving as negative and positive controls, respectively. Although autoantibody levels in *XCR1*^*iCre*^*Cldn1*^*fl/fl*^ mice did not reach those detected in *OVA*^*+*^*Cldn1*^*−/−*^ chimeras, they were significantly elevated compared with *Cldn1*^*fl/fl*^ mice (Fig. S5, H and I). In particular, increased autoantibody reactivity was observed against composite tissue and stomach with a notable trend toward higher levels in the liver, while kidney-specific autoantibodies remained unchanged. Consistent with these findings, *XCR1*^*iCre*^*Cldn1*^*fl/fl*^ mice also displayed an increased colon weight-to-length ratio compared with *Cldn1*^*fl/fl*^ controls (Fig. S5, J and K), recapitulating alterations observed in the competitive chimera setting. However, no statistically significant increase in premature mortality was detected between *XCR1*^*iCre*^*Cldn1*^*fl/fl*^ and *Cldn1*^*fl/fl*^ mice up to 52 wk of age (Fig. S5 L). Collectively, these results indicate that the DC1-intrinsic expression of Claudin 1 is essential for the prevention of multiorgan autoimmunity.

## Discussion

In this study, we identified the function of the tight junction protein, CLAUDIN 1, in antigen transfer to thymic DC1 lineage and its maturation. We annotated the phenotypic heterogeneity of this lineage and performed tracing experiments that confirmed the immature DC1 transition to early mature aDC1a and subsequently to late mature aDC1b. A DC1 lineage–specific knockout of the *Cldn1* gene showed that Claudin 1 is critical for the maturation of aDC1a and aDC1b. Since CAT-experienced DC1 lineage maturation was accompanied by the upregulation of cholesterol efflux–associated genes, we suggest that CAT is responsible for the homeostatic maturation of DC1s. We also detected the highest expression of CLAUDIN 3, the binding partner of CLAUDIN 1, on TRA-expressing mTECs, which lends credence to the phenomenon of their preferential pairing with XCR1^+^ and XCR1^−^ aDCs (Vobořil et al., 2022). The importance of Claudin 1 was confirmed by light sheet fluorescence microscopy, which illustrated that it ensures optimal positioning of DC1s within the mTEC network. By comparing the transcriptomes of Claudin 1–sufficient and Claudin 1–deficient DC1 lineages, we showed that Claudin 1 is critical for the expression of *Ctse* and other genes involved in MHCII presentation. Consequently, we ablated MHCII in DC1 lineage and detected perturbed DC1 maturation and Treg selection. Finally, we generated a novel *Defa6*^*iCre*^*R26*^*TdT-OVA*^ mouse model with OVA neo-self-antigen that mimicked TRA expression and showed that Claudin 1 deficiency in the DC1 lineage diminished clonal deletion of OVA-specific T cells, as well as their selection into Tregs. Consistent with impaired central tolerance, we detected high serum titers of autoantibodies against several tissues, increased frequency of effector CD4^+^ T cells and Tregs, and symptoms of colitis. This break in tolerance correlated with the shortened lifespan of these animals. In aggregate, we uncovered a novel molecular mechanism that employs Claudin 1 as an essential molecule in CAT and maturation of cells of the DC1 lineage, which in turn provides an antigen-presenting network for proper thymic selection of TRA-specific T cells.

The compilation of our data consolidates the current knowledge regarding the classification of aDC subsets and their maturation path. The scRNAseq thymus atlas developed by Park and colleagues divided aDCs into two conventional DC lineages: *Xcr1*^*+*^ aDC1 and *Sirpa*^*+*^ aDC2, along with an aDC3 subset, which exhibited dramatically reduced or a lack of expression of *Sirpa* and *Xcr1*, respectively, as well as other lineage-specific markers (Park et al., 2020). Recently, Bosteels and colleagues described two maturation states of splenic aDC1s, early and late, the latter also showing decreased levels of *Xcr1* (Bosteels et al., 2023; Bosteels and Janssens, 2025). Our scRNAseq confirmed that both the DC1 and DC2 lineages contain two maturation states. Focusing on the DC1 lineage, we showed that DC1 give rise to aDC1a, which are the predecessors of aDC1b. Along with maturation from the a to b state, accompanied by the gradual diminishment of *Sirpa* or loss of *Xcr1*, we observed upregulation of two genes, *Cd81* and *Il7r*, that are affiliated with DC1 and DC2 lineages, respectively. Therefore, these genes can be used as surrogate markers for the late mature stages, aDC1b and aDC2b, when *Xcr1* and *Sirpa* are absent or downregulated, respectively. In contrast to our lineage tracing experiments, attempts to track the development of either thymic or extrathymic DCs failed to detect XCR1^−^ aDC1b inside the DC1 lineage (Ardouin et al., 2016; Bosteels et al., 2023). Therefore, in the thymus, aDC1a represents early mature XCR1^+^ DC1s, which in the past were referred to as mDC1, CCR7^+^ DC1, mregDC1, or aDC1, while aDC1b represents late mature XCR1^−^ DC1s (Ardouin et al., 2016; Maier et al., 2020; Park et al., 2020; Breed et al., 2022; Vobořil et al., 2022; Bosteels et al., 2023; Ashby et al., 2024).

We have shown that thymic DC1 homeostatic maturation is similar to what has been described in splenic DC1s (Bosteels et al., 2023; Ardouin et al., 2016). Surprisingly, homeostatic maturation of DCs, unlike immunogenic maturation, does not involve the engagement of pattern recognition receptors (Bosteels and Janssens, 2025), yet it leads to nearly the same changes in the transcriptome of DCs regardless of the type of the lymphoid tissue, i.e., thymus, spleen, or lymph nodes (Ardouin et al., 2016). However, DC1 homeostatic maturation differs in transcriptomic changes related to genes involved in fat metabolism and interferon sensing (Ardouin et al., 2016; Bosteels et al., 2023; Ashby et al., 2024). Consistent with this notion, we detected the upregulation of cholesterol efflux–associated genes in CAT-experienced DC1s, which accompanies the maturation process. This result is in agreement with a scenario where mTEC-derived cholesterol-containing apoptotic bodies induce the liver X receptor pathway, driving the efflux of cholesterol which induces homeostatic maturation of the thymic DC1 lineage, analogous to what has been proposed in splenic DC1 maturation (Bosteels et al., 2023). In this context, a complementary thymus-specific mechanism that is responsible for DC1 maturation, which depends on type III interferon sensing, has been recently reported (Ashby et al., 2024). In contrast to DC1s, the DC2 lineage was found to be nonresponsive to apoptotic cell engulfment in terms of its maturation (Bosteels et al., 2023). Instead, thymic DC2s display a type 2 cytokine gene expression signature requiring IL-4R signaling to become mature and engage in clonal deletion (Breed et al., 2022).

While some maturation drivers are DC lineage–specific, homeostatic maturation of both thymic DC1 and DC2 requires the presence of T cells, TCR-MHCII interactions, and, to a lesser extent, CD40 signaling (Oh et al., 2018). Consistent with this, we demonstrated that DC1 lineage–specific ablation of MHCII leads to an accumulation of immature DC1s and a paucity of aDC1 cells. Since we found that Claudin 1 deficiency leads to the abrogated expression of *Ctse* and downregulated expression of *Cd207* in addition to other genes involved in the MHCII pathway, we propose that the involvement of Claudin 1 in antigen presentation is a plausible mechanism that regulates DC1 maturation. Taken together, we suggest that thymic DC maturation is a multistep process that, in the case of the DC1 lineage, requires the engulfment of apoptotic bodies, type III interferon stimulation, and antigen presentation to T cells.

As alluded to in the Results section, we have shown that Claudin 1 is required for the juxtaposition of DC1s to mTECs within the epithelial network. This positioning may represent an analogous scenario that has been observed in the gut or skin where DCs cross the epithelial barrier in a Claudin 1–dependent manner to capture and subsequently present antigens (Rescigno et al., 2001; Kubo et al., 2009). Our observation of the highest expression of Claudin 3, which is the ligand of Claudin 1, on mTECs that produce Aire-dependent TRAs supports this hypothesis. Interestingly, recently discovered mimetic cells (Michelson et al., 2022; Givony et al., 2023), which express high levels of TRAs but may possess only a limited antigen presentation capability (Březina et al., 2022), also display high levels of Claudin 3. This predisposes mimetic cells, in addition to mTEC^HI^ cells, to be prime targets for the Claudin 1^+^ DC1 lineage to acquire TRAs via CAT. Other CAT-associated molecules identified in our scRNAseq screening fit within this framework including the integrin, CD103 (encoded by *Itgae*), which mediates adhesion of DCs to the epithelium (Del Rio et al., 2010). To accomplish CAT, molecules such as scavenger CD36 are then required for the acquisition of mTEC antigens. Considering that the execution of CAT is likely the result of cooperative action of several molecular determinants that are expressed on DC1s or by their mTEC-interacting partners, we propose that the loss of function of any of these determinants will be at least partially compensated by other molecules. The ablation of Claudin 1 in the DC1 lineage had no gross effect on the efficiency of CAT but severely impaired the frequency of CAT-experienced aDC1b. This can be explained by two possible scenarios: (1) with respect to CAT, the absence of Claudin 1 can be readily substituted by other determinants or (2) Claudin 1 is necessary only for the maturation of the DC1 lineage, which is preceded by CAT.

Unexpectedly, Claudin 1 deficiency resulted in the profound reduction of only TdTOMATO^+^ aDC1b, even though Claudin 1 expression was low in this subset. We presumed that Claudin 1 deficiency should affect aDC1a since this subset expressed the highest levels of Claudin 1. Even though we annotated aDC1a and aDC1b as separate subsets, the developmental process from aDC1a to aDC1b represents a continuum of maturation states. Notably, the observation that Claudin 1–deficient DC1 lineage tends to accumulate TdTOMATO^+^ cells in the aDC1a state, which likely reflects a stalled maturation, suggests that Claudin 1 is critical for the transition from aDC1a to aDC1b. In addition, since the cellularity of Claudin 1–deficient TdTOMATO^+^ aDC1a compared with its Claudin 1–sufficient counterpart in the same competitive BM chimeric mice was decreased, it is plausible that Claudin 1 influences not only maturation but also the survival rate of this subset. Regardless of the mechanism through which Claudin 1 regulates DC1 maturation, the severe reduction of aDC1b subset is one of the crucial findings of this study. Since aDC1b is a fully matured subset, which specializes in antigen presentation, its significantly reduced numbers can explain how defective DC1 maturation leads to a break in central tolerance.

The Claudin 1–dependent juxtaposition of DC1 to mTEC^HI^ may also explain the role of Claudin 1 in DC1 maturation. Notably, Claudin 1 may act as a sensor that stimulates the acquisition of TRAs. In this scenario, activation of Claudin 1 via binding to mTEC^HI^ may couple CAT with efficient antigen presentation via upregulated expression of genes such as *Ctse*. In turn, this would lead to DC1 maturation via enhanced TCR-MHCII interactions with T cells. The proximity of DC1 to mTEC^HI^ may also fuel DC1 maturation by an exposure to type III interferons (whose expression in the thymus is restricted to mTEC^HI^ cells [Ashby et al., 2024]) and CAT (Bosteels et al., 2023). It is important to note that CAT has the potential to promote DC1 maturation, regardless of Claudin 1, by, for example, stimulating cholesterol metabolism (Bosteels et al., 2023). Alternatively, the role of Claudin 1 in DC1 maturation may be explained by previous studies, which showed that the Claudin family of proteins interacts with several classes of nontight junction molecules such as tetraspanins (Van Itallie and Anderson, 2013). It is of particular interest that Claudin 1 interacts with the tetraspanin family member CD81, which is a molecular sensor of cholesterol (Harris et al., 2008; Palor et al., 2020) and is co-expressed with Claudin 1 in aDC1a population. As we previously noted, cholesterol sensing during apoptotic cell acquisition drives homeostatic maturation of the DC1 lineage (Bosteels et al., 2023). Our finding showing the gradual expression of *Cd81* during DC1 maturation suggests that Claudin 1 may drive the maturation of CAT-experienced DC1 through its interaction with CD81 and cholesterol sensing.

We have shown that in the absence of Claudin 1 in the thymic DC1 lineage, the immune periphery exhibited an elevated frequency of self-reactive conventional T cells and a reduced frequency of Tregs, attesting to the leakiness of central tolerance. In fact, mice carrying Claudin 1–deficient DC1s manifested an autoimmune phenotype, which closely mimicked the symptoms of other mouse models that suffer from insufficient presentation of TRAs in the thymus, such as *Aire*^−/−^ mice, which are characterized by high autoantibody titers, multiorgan autoimmunity, and shortened life expectancy (Jiang et al., 2005; Abramson and Husebye, 2016). In general, dysregulation of Claudin 1 expression contributes to numerous autoimmune diseases including rheumatoid arthritis, type 1 diabetes, multiple sclerosis, or inflammatory bowel disease (Sapone et al., 2006; Weber et al., 2008; Mandel et al., 2012; Tajik et al., 2020). While *Cldn1*^*−/−*^ mice die within the first day of life due to transepidermal water loss (Furuse et al., 2002), the Claudin 1 knockdown showed a disintegration of the epidermis that resembled atopic dermatitis (Tokumasu et al., 2016). Strikingly, the phenotype of mice deficient for *Ctse*, the expression of which was found absent in the Claudin 1–deficient DC1 lineage, is also demonstrated by atopic dermatitis (Tsukuba et al., 2003). As previously mentioned, Claudin 1 is expressed by peripheral DCs that home to epithelial organs, including the skin (Rescigno et al., 2001; Farache et al., 2013; Kubo et al., 2009; Sung et al., 2006). Thus, although there is a consensus that abnormal Claudin 1 expression in the epithelium leads to loss of barrier integrity and subsequent breakdown of peripheral tolerance, the absence of Claudin 1 on peripheral DC1s may also contribute to tolerance defects.

It is critical to note that using the tools available to us, we were unable to investigate whether the observed autoimmune manifestations in our Claudin 1–deficient models were solely caused by the central tolerance breakdown or whether a breakdown of peripheral tolerance also contributed. In addition, based on our data, we were unable to determine the reason(s) for the observed central tolerance defects. Likely causes include a defect in CAT, DC1 maturation, or DC1 antigen presentation, as well as their mutually additive or synergistic effects. In this context, a critical factor may be that Claudin 1–deficient DC1s lacked cathepsin E and therefore, given its high substrate specificity (Arnold et al., 1997), were unable to present a specific set of self-peptides that would be recognized by a portion of developing self-reactive T cells. Regardless of the molecular mechanism involved, we propose that Claudin 1 deficiency may lead to autoimmunity through the loss of mature, CAT-experienced, antigen presentation–efficient thymic DC1s, which limits the ability of central tolerance to delete self-reactive T cells or convert them into Tregs. Collectively, Claudin 1 dysregulation likely leads to autoimmunity both indirectly through the loss of the epithelial barrier integrity and directly through the failure of the DC1 lineage to mature and to purge self-reactive T cells.

## Materials and methods

### Mice

All mice used in this study were on the full *C57BL/6J (B6)* background, except for *Aire*^−/−^ mice that were on the full *BALB/c* background and bred under SPF conditions at the animal facility of the Institute of Molecular Genetics of the Czech Academy of Sciences (IMG) and University of Birmingham, Biomedical Services Unit, Birmingham, UK. Experimental procedures with mice were approved by the ethical committee of the IMG, Birmingham Animal Welfare and Ethical Review Board, and UK Home Office. Mice were fed by standard rodent high-energy diet and given reverse osmosis–filtered water ad libitum. Mice were bred under light/dark cycle that oscillated every 12 h and in constant temperature and humidity of 22 ± 1°C and 55 ± 5%, respectively. *B6*, *Foxn1*^*Cre*^ (B6(Cg)-*Foxn1*^*tm3(cre)Nrm*^/J; #018448) (Gordon et al., 2007), *Ly5.1* (B6.SJL-*Ptprc*^*a*^*Pepc*^*b*^/BoyJ; #002014) (Janowska-Wieczorek et al., 2001), *I-Ab*^*fl/fl*^ (B6.129X1-*H2-Ab1*^*b-tm1Koni/*^J; #013181) (Hashimoto et al., 2002), *Rag1*^*−/−*^ (B6.129S7-*Rag1*^*tm1-Mom*^/J; #002216) (Mombaerts et al., 1992), and *OTII* (B6.Cg-Tg(TcraTcrb)425Cbn/J; #004194) (Barnden et al., 1998) mice were purchased from the Jackson Laboratories. *R26*^*TdTOMATO*^ mice (B6;129S6-*Gt(ROSA)26Sor*^*tm14(CAG-tdTomato)Hze*^/J; #007908) (Madisen et al., 2010) were provided by V. Kořínek (IMG, Prague, Czech Republic). *Defa6*^*iCre*^ mice (Adolph et al., 2013) were kindly provided by R.S. Blumberg (Division of Gastroenterology, Department of Medicine, Brigham and Women’s Hospital, Harvard Medical School, Boston, MA, USA). *XCR1*^*iCre*^ mice (Wohn et al., 2020) were kindly provided by B. Malissen (Centre d’Immunologie de Marseille-Luminy, Aix Marseille Universite´, Inserm, CNRS, Marseille, France). *Cldn1*^*fl/fl*^ mice (Tokumasu et al., 2016) were kindly provided by S. Tsukita (Advanced Comprehensive Research Organization, Teikyo University, Tokyo, Japan). *Adig*^*GFP*^ (Gardner et al., 2008) and *Aire*^*−/−*^ (Ramsey et al., 2002) mice were kindly provided by L. Klein (Ludwig Maximilian University, Munich, Germany). To harvest murine tissues, mice were euthanized by cervical dislocation at 4–7 wk of age, except for BM chimera experiments, where BM was transplanted into sublethally irradiated mice at 5–8 wk of age. These mice were euthanized 5–6 wk after BM transplantation, except for the mice subjected for the analysis of autoimmunity symptoms, which were culled 30–40 wk after BM transplantation or used for the survival curve experimentation. In addition, *Aire*^*−/−*^ mice were euthanized at 30 wk of age. The average age of nonchimeric *XCR1*^*iCre*^*Cldn1*^*fl/fl*^ and *Cldn1*^*fl/fl*^ mice subjected for the analysis of autoimmunity symptoms was ∼35 wk. In all individual experiments, littermates were used regardless of their sex and caging. BM donors and mice used for scRNAseq and bulkSeq were females.

### *R26* ^*TdT-OVA*^ mouse model

To generate mice with inducible TdTOMATO-OVA (TdT-OVA) expression, we designed and synthesized a plasmid vector (GenScript) for site-specific integration into a mouse Rosa26 (R26) locus. This vector includes a CAG promoter, a loxP-STOP-loxP cassette, the TdT-OVA transgene, a Woodchuck hepatitis virus posttranscriptional regulatory element, and a bovine growth hormone polyadenylation signal. We flanked the knock-in sequence with R26 homology arms (Kasparek et al., 2014) and gRNA target sequences (5′-CTC​CAG​TCT​TTC​TAG​AAG​ATG​GG-3′), to facilitate the efficiency of site-specific integration (Yao et al., 2017). Using the online software CRISPOR Design Tool (https://crispor.gi.ucsc.edu/; Concordet and Haeussler, 2018), we designed a R26 targeting gRNA (5′-CTC​CAG​TCT​TTC​TAG​AAG​AT-3′). For pronuclear microinjections, we combined the targeting vector with gRNA and Cas9 protein and then introduced them into C57BL6n-derived zygotes as previously described (Kasparek et al., 2014). Founder animals carrying site-specific insertion were identified using PCR with primers flanking homology arms. The full-length knock-in sequence was verified by Sanger sequencing. The expression of TdT-OVA protein by *R26*^*TdT-OVA*^ mice crossed to *Itgax*^*Cre*^ (Caton et al., 2007), *Foxn1*^*Cre*^, or *Defa6*^*iCre*^ mice was verified by flow cytometry. Specifically, we tested the presence of OTII peptide in Figs. 6 and S4, OTI peptide by SIINFEKL staining and proliferation assay of OTI TCRtg T cells, and TdTOMATO expression and its CAT by crossing *R26*^*TdT-OVA*^ mice to *Foxn1*^*Cre*^ and *Defa6*^*iCre*^ strains.

### Cell isolations

Thymi were isolated using forceps, cut into 10–15 pieces, and digested with an enzymatic cocktail of 0.1 mg/ml Collagenase D (Roche) and DNase I (40 U/ml; Roche) dissolved in RPMI/3% FBS medium. Note that pieces of each thymus were put into 1 ml of enzymatic cocktail in a 1.5-ml Eppendorf tube. To isolate TECs, 0.1 mg/ml Dispase II (Gibco) was added to the enzymatic cocktail. To complete digestion, enzymatic cocktails containing thymi were put into thermoshaker and incubated for ∼80 min at 37°C while shaken at 800 rpm. After incubation, nondigested thymic pieces were pipetted up and down several times using a cut pipette tip until the solution was homogeneous and then filtered into a 15-ml Falcon tube. To stop the enzymatic reaction, each thymic solution was washed with 2 ml of ice-cold 3% FBS and 2 mM EDTA solution in PBS. Then, thymi were spun down (4°C, 400 × *g*, 10 min). In the case of T-cell isolation, pellets were resuspended in 1 ml of ACK lysis buffer, incubated for 3 min, washed with 14 ml of 3% FBS and 2 mM EDTA solution in PBS, and spun down (4°C, 400 × *g*, 10 min). 1/10 of the pellet was used for later analysis. In the case of thymic myeloid APCs or TEC isolations, Percoll (Cytiva) enrichment was conducted by resuspending the pellets in 2 ml PBS, underlaid with 2 ml of 1.065 g/ml Percoll and then 2 ml of 1.115 g/ml Percoll to create three separate layers. Next, the samples underwent gradient centrifugation (4°C, 1,500 × *g*, 30 min, w/o break and acceleration). After centrifugation, two cell layers were formed. The bottom layer consisted of smaller cells such as T cells and erythrocytes, and the upper layer consisted of myeloid APCs and TECs. The cells from the upper layer were gently transferred into 10 ml of 3% FBS and 2 mM EDTA solution in PBS and spun down (4°C, 300 × *g*, 10 min). The resulting pellet was used for further analysis. In the case of isolation of splenic T cells or T cells from lymph nodes, the same approach that was used for the isolation of thymic T cells was applied. Note that one-fifth of the spleen was used for cell isolation and such one-fifth was cut into ∼10 pieces. Lymph nodes were opened using a 26G needle. The requirements for the isolation of cells designated for scRNAseq are described in its dedicated paragraph.

### Flow cytometry analysis

To stain cell surface markers for flow cytometry (FACS) analysis, cells were incubated with antibodies or other staining reagents at 4°C in the dark for 20–30 min. Note that biotin-conjugated antibodies were stained prior to staining with fluorochrome-conjugated streptavidin and antibodies against other surface markers at 4°C in the dark for 20–30 min. In the case of anti-CCR7 antibody (BioLegend) staining, incubation was conducted on a thermoshaker at 37°C, 800 rpm for a minimum of 30 min prior to all other staining incubations. After each incubation, cells were washed using 1 ml of 3% FBS and 2 mM EDTA solution in PBS and spun down (4°C, 300 × *g*, 10 min). Note that in the case of T-cell staining, the centrifugation force was 400 × *g*. To stain intracellular markers, cells were fixed using a Foxp3 staining kit (Thermo Fisher Scientific) for 30 min after surface staining according to the manufacturer’s protocol. Fixed cells were incubated with primary antibodies for 30 min at room temperature (RT), and in the case of unconjugated primary antibody staining, an additional 15-min staining at RT with secondary antibodies was conducted. After each incubation, cells were washed using 10x diluted permeabilization buffer (Thermo Fisher Scientific) and spun down (4°C, 500 × *g*, 10 min). Dead cells were excluded using either Hoechst 33258 (Sigma-Aldrich) or fixable viability dye eFluor 506 (eBioscience). FACS analysis was performed using FACSymphony A5, LSRFortessa, FACSDiva software, and FlowJo v10 software (BD). A list of antibodies and other staining reagents can be found in Table S1.

### Single-cell RNA sequencing

To perform scRNAseq of thymic myeloid APCs, thymi from 6-wk-old *Foxn1*^*Cre*^*R26*^*TdTOMATO*^ mice were enzymatically digested as described (see Cell isolations). Importantly, isolated cells were not subjected to Percoll enrichment but were resuspended in a cocktail of anti-CD11c and anti-CD11b antibodies both conjugated with biotin, stained on ice for 25 min, washed with 3% FBS and 2 mM EDTA solution in PBS, and then spun down (4°C, 300 × *g*, 10 min). Next, cells were stained with anti-biotin magnetic beads (Miltenyi) according to the manufacturer’s protocol and CD11c^+^ and CD11b^+^ cells were MACS-enriched using QuadroMACS (Miltenyi). Enriched cells were stained with fluorochrome-conjugated streptavidin on ice for 15 min, washed with 3% FBS and 2 mM EDTA solution in PBS, and then spun down (4°C, 300 × *g*, 10 min). Next, ∼1 × 10^5^ of Streptavidin^+^ cells were sorted using a BD Influx cell sorter (BD) from a pool of three female littermate thymi. Importantly, half of the cells were sorted as TdTOMATO^+^ (CAT-experienced cells) and the other half as TdTOMATO^−^ (CAT-inexperienced cells) into separate collection tubes to prepare two individual scRNAseq libraries (samples). To check that viability of sorted cells was >90%, an automated TC20 cell counter (Bio-Rad) was used. scRNAseq libraries were prepared using a Chromium controller and the Chromium Next Gen Single Cell 3′ Reagent Kit version 3.1 (both from 10X Genomics) according to the manufacturer’s protocol targeting 4,000 cells per sample, i.e., 4,000 of TdTOMATO^+^ and TdTOMATO^−^ cells sequenced. The quality and quantity of the resulting cDNA and libraries were determined using Agilent 2100 Bioanalyzer (Agilent Technologies). The sample libraries were sequenced in a single run of NextSeq 500 instrument (Illumina) using a high-output kit with mRNA fragment read length of 56 bases. We used 10X Genomics Cell Ranger software suite (version 3.1.0) to quantify gene-level expression based on GRCm38 assembly (Ensembl annotation version 98) (Yates et al., 2020). See the Data availability section below for the link and accession number to the scRNAseq data.

### Bioinformatics analysis of scRNAseq

For bioinformatics analysis of 10x scRNAseq data, we used a standard Seurat (v 4.0.2) pipeline, which was performed in R v 4.0.2 (R Core Team 2020) in a similar setting as done previously (Brabec et al., 2024). In brief, 10x Cell Ranger raw read counts were used as an input. Cells were filtered to obtain those containing at least 1,000 detected features (genes) and <20% of mitochondrial RNA read counts. Samples were then pooled and processed together. Cell types were annotated using a combination of clustering and canonic cell type–specific marker gene expression profile. If a cluster contained a mixed population, subclustering was performed. Afterward, the cell type definition dataset was filtered to obtain clusters containing only conventional DCs and monocyte/macrophage lineages. This filtered dataset was again clustered, and sublineage cell types were defined using the same method as noted above. Seurat embedded differential expression analysis was used to define genes whose expression accompanies CAT in individual cell types, as well as additional cell type markers.

### BM chimeras

To prepare BM chimeras, femurs and tibias of euthanized BM donors were isolated, cleaned of surrounding tissues, and cut at both ends. BM was flushed out from the bones using PBS and syringe with 26G needle into a 15-ml Falcon tube. Isolated BM was spun down (4°C, 400 × *g*, 10 min). The obtained pellets were resuspended with 1 ml of ACK lysis buffer, incubated for 3 min, washed with 14 ml of 3% FBS and 2 mM EDTA solution in PBS, and spun down (4°C, 400 × *g*, 10 min). Recipient mice were sublethally irradiated with 6 Gy, and each mouse received 2 × 10^6^ of isolated BM cells through the tail vein. Importantly, prior to the transplantation, BMs were mixed at a 50:50 (Figs. 3, 4, and 5) or 45:45:10 ratio (Figs. 6 and 7). After transplantation, the mice were monitored daily for signs of infection/wasting following irradiation. To protect the mice against infection, we supplemented their water with 2 ml/100 ml of gentamicin (Aagent) for 2 wk. The efficiency of reconstitution of mixed BM chimeras is shown in Fig. 4 E; Fig. 5 A; Fig. S3, D and E; and Fig. S4, F and G.

### BrdU lineage tracing

To trace DC1 lineage, WT *Ly5.1* mice were injected with 1.5 mg of BrdU i.p. and culled at 1, 2, 3, 4, 5, or 7 day(s) after BrdU administration. Thymi of culled mice were enzymatically digested, and thymic DCs were isolated. To stain BrdU^+^ thymic DCs, an established protocol was used (Cosway et al., 2018). Briefly, thymic DCs were resuspended in 100 μl of BD fix/perm (BD) and incubated for 30 min at 4°C. Next, cells were washed in 1ml Perm/Wash (BD) and spun down (4°C, 500 × *g*, 10 min). The pellet obtained was resuspended in 100 μl of BD Cytoperm Buffer Plus (BD), incubated for 10 min on ice, and washed. Afterward, another round of fixation in 100 μl of BD fix/perm for 5 min on ice was conducted followed by washing. Next, cells were treated with DNase I (1 mg/ml) for 45 min at 37°C and washed. Then, DNase-treated cells were stained with anti-BrdU FITC antibody diluted 1:100 in 100 μl Perm/Wash buffer for 20 min at RT. After staining, cells were washed and subjected to FACS analysis.

### Light sheet fluorescence microscopy

For the segmentation, distance calculation, and visualization of thymic DC1s and mTECs, light sheet fluorescence microscopy was used. Thymi of *Adig*^*GFP*^ chimeras possessing mixed BMs were harvested 6 wk after BM transplantation. Thymic lobes were separated, and one was used for flow cytometry analysis of BM reconstitution. Thymic lobes used for microscopy were first fixed overnight at 4°C in 3.8% paraformaldehyde. After thorough washing, the samples were cleared using a modified CUBIC protocol (Pačes et al., 2022, 2023). In the first step, the samples were cleared for 5 days at 37°C in CUBIC1 solution (35 wt% dH_2_O, 25 wt% urea, 25 wt% N,N,N′,N′-Tetrakis(2-hydroxypropyl) ethylenediamine (4NTEA), 15 wt% Triton X-100). The cleared samples were rinsed for one h in CUBIC wash solution (0.5% BSA, 0.01% sodium azide, 0.01% Triton X-100 in PBS) three times. Subsequently, the samples were incubated in CUBIC 2 clearing solution (23.4 wt% dH_2_O, 22.5 wt% urea, 9 wt% triethanolamine [TEA], 45 wt% sucrose, 0.1% [vol/vol] Triton X-100) for 3 days to achieve refractive index matching. Analogical medullary regions of the same size were then imaged at RT using a Zeiss Z.1 light sheet microscope and Zeiss light sheet fluorescence microscopy clearing 10×/0.2, detection objective (Objective Clr Plan-Neofluar 20×/1.0 Corr nd = 1.45 M32 85mm) equipped with a 1.45 RI clearing chamber and Zeiss sCMOS pco.edge 5.5m Camera Mic-System. Zen Black edition LS was used for the image acquisition. A total of three channels were acquired: green (excitation: 488 nm; detection: 498 nm) for GFP signal detection, red (excitation: 561 nm; detection: 571 nm) for TdTOMATO signal detection, and a cross-channel (excitation: 488 nm; detection: 571 nm) to capture autofluorescence. This autofluorescence helped to distinguish positive signals in the two specific channels during subsequent steps. The raw data were first 3D-deconvolved using Huygens Professional software (Pačes et al., 2023) and subsequently analyzed in Arivis 4D (version 4.2.) with the following steps. First, voxel training was performed for the machine learning segmenter using all three channels until the model reliably recognized DC1s and mTEC clusters from the background. Subsequently, the TdTOMATO^+^ DC1s and GFP^+^ mTEC clusters were segmented. In the second step, splitting was performed for DC1s with a sensitivity of 57.33%. In the third and fourth steps, both categories were filtered and all artifacts and fragments smaller than 400 μm^3^ were removed. Subsequently, the minimum distances between each DC1 and the nearest mTEC cluster were measured using the Distances module. Finally, three expansions of the mTEC clusters were performed sequentially in the Compartments module, by 5, 25, and 50 μm, and the percentage of DC1s from total in each of the expanded clusters was counted.

### Bulk RNA sequencing

To perform bulkSeq, thymic DCs were isolated from three female competitive BM chimeras (Fig. 3 D) 7 wk after BM transplantation using a standard cell isolation protocol for flow cytometry. Isolated cells were FACS-sorted using an Aria sorter (BD). Specifically, we sorted 3,000 DC1, 1,500 aDC1a, and 1,500 aDC1b of both Cldn1^+/+^ and Cldn1^−/−^ origins from three biological replicates. We pooled the cells that shared the same origin, resulting in six samples in total. Single-cell suspensions were sorted directly into Lysis/Binding Buffer (Invitrogen) and immediately frozen on dry ice. RNA was isolated using Dynabeads (Invitrogen) according to the manufacturer’s protocol. Sequencing libraries were prepared using the MARS-seq protocol, as described previously (Jaitin et al., 2014). Libraries were sequenced with NextSeq P2 XLEAP-SBS Reagent Kit on a NextSeq 2000 sequencer (Illumina). Differential gene expression analysis was performed using the UTAP pipeline (Kohen et al., 2019). Log_2_ fold changes generated from this pipeline were used as input for GSEA performed with clusterProfiler (v 4.12.0) in R (v 4.4.0) and visualized using enrichplot (v 1.24.0). Gene ontology terms were used as the gene set resource. See the Data availability section below for the link and accession number to the bulkSeq data.

### Autoantibody detection

Autoantibodies were detected in serum samples using a NovaLite rat liver, kidney, and stomach multicomposite kit: “Composite tissue slides” (Innova Diagnostics). Briefly, blood was drawn from a facial blood vessel into a microtube precoated with 0.2 μl 0.5 M EDTA and spun down (4°C, 2,000 × *g*, 15 min) to obtain the sera. Composite tissue slides were incubated with 1/40 sera at RT according to an established protocol (Alawam et al., 2021), followed by detection with goat anti-mouse IgG (H+L) Alexa 555 (Thermo Fischer Scientific). Composite tissue slides were stained with DAPI and mounted using AD Mount mounting media (ADVI s.r.o.). Images were acquired at RT using a DM6000 microscope (Leica Microsystems) with HCX PL APO 40×/0.75 DRY PH2; FWD 0.28 CG 0.17 objective lens; and Leica DFC 9000—monochromatic sCMOS camera. LAS X 64-bit software (Leica) was used for image acquisition. Quantification of autoantibodies was performed by measuring the mean fluorescence intensity (MFI) at selected regions of interest corresponding to specific tissue areas using the ImageJ program (NIH). Data in Fig. 7 B were normalized according to the MFI background of a secondary antibody staining in each experiment.

### Analysis of autoimmunity in the intestine

For the counting of PCs, mouse terminal ilea were isolated, cleared of feces, and fixed in 4% paraformaldehyde overnight. They were then placed in 70% ethanol overnight, dehydrated, and embedded in paraffin using the Leica HistoCore Pegasus Tissue Processor, and subsequently the Leica HistoCore Arcadia. Embedded ilea were longitudinally cut into 10-μm sections. Prior to staining, sections were blocked with 10% bovine serum albumin in PBS. To visualize PC, paraffin sections were stained with polyclonal anti-lysozyme antibody (Agilent/Dako; host: rabbit). Then, goat anti-rabbit IgG (H+L) Alexa 555–conjugated secondary antibody (Thermo Fisher Scientific) was applied followed by DAPI staining. The sections were mounted in Vectashield (Vector Labs) and imaged at RT using a Leica DMi8 microscope with a 20× magnification objective lens and Zyla CMOS camera (Andor). LAS X 64-bit software (Leica) was used for both acquisition and analysis. The average number of PCs per crypt was determined as previously described (Brabec et al., 2023). For analysis of the colon weight/length ratio, mouse colons were isolated along with the cecum. The isolated tissue was stretched, and the length from the cecum to rectum was measured. The colons were then separated from the cecums, cleaned of feces, and weighed. The ratio was calculated by dividing the weight of the colon (mg) by the length of the colon (mm).

### Statistical analysis

Statistical analysis and graphs were generated using Prism 10.5.0 software (GraphPad), except for scRNAseq, which was analyzed using the Seurat package, R v 4.0.2 (R Core Team 2020), and bulkSeq, which was analyzed using RStudio (R v 4.4.0 and 4.5.1.). When comparing two experimental groups, unpaired or paired Student’s *t* test was used. One-way ANOVA with Tukey’s multiple comparisons test was used to compare three or more experimental groups. When pairing between samples was applicable, repeated-measures one-way ANOVA with Tukey’s multiple comparisons test was used. Survival curve statistics was analyzed using the log-rank (Mantel–Cox) test. In the scatter plots and BrdU lineage tracing plot, the mean ± SEM is shown. Median and quartiles are shown for some of the violin plots. Sample sizes, experimental replicates, and additional information such as type of normalization are provided in the figure legends. If P ≤ 0.05, it is considered statistically significant. In certain cases where we refer to a possible statistically significant trend, the abbreviation “ns” has been replaced by the exact P value in the figures. Light sheet fluorescence microscopy, autoantibody detection, and PC counting experiments were imaged and analyzed blinded by a source unfamiliar with the genotype/phenotype of the mice. Mice and tissue samples were excluded from the analysis if BM reconstitution was insufficient, or cell isolation was suboptimal. The pipelines of scRNAseq and bulkSeq analyses are described in the sections dedicated to these methods.

### Online supplemental material

Fig. S1 shows scRNAseq of thymic myeloid APCs. Fig. S2 shows the expression of marker and antigen presentation–associated genes by thymic DCs. Fig. S3 shows mouse models used to study the role of Claudin in CAT. Fig. S4 shows MHCII-mediated antigen presentation and Claudin 1 expression by DC1 lineage cells are important for the establishment of central tolerance. Fig. S5 shows autoimmune manifestations of Claudin 1–deficient mice. Table S1 shows list of antibodies. Data S1 shows general source data. Data S2 shows marker genes of thymic myeloid cell subsets related to Fig. S1 D. Data S3 shows marker genes of aDC subsets related to Fig. S1 E. Data S4 shows DEGs between TdTOMATO^+^ and TdTOMATO^−^ DC1 related to Fig. 1 D. Data S5 shows bulkSeq data from Fig. 5.

## Supplementary Material

Table S1 shows list of antibodies.

Data S1 shows general source data.

Data S2 shows marker genes of thymic myeloid cell subsets related to Fig. S1 D.

Data S3 shows marker genes of aDC subsets related to Fig. S1 E.

Data S4 shows DEGs between TdTOMATO^+^ and TdTOMATO^−^ DC1 related to Fig. 1 D.

Data S5 shows bulkSeq data from Fig. 5.

## Data Availability

The sequencing data are available in the BioStudies database under the accession number E-MTAB-14319 (scRNAseq) (https://www.ebi.ac.uk/biostudies/arrayexpress/studies/E-MTAB-14319) and E-MTAB-15468 (bulkSeq) (https://www.ebi.ac.uk/biostudies/arrayexpress/studies/E-MTAB-15468). Source data files for all figures and supplementary figures can be found in Data S1. All data needed to evaluate the conclusions of this study are present in the paper or in Supplementary materials.
